# The Protein Kinase A-Dependent Phosphoproteome of the Human Pathogen Aspergillus fumigatus Reveals Diverse Virulence-Associated Kinase Targets

**DOI:** 10.1128/mBio.02880-20

**Published:** 2020-12-15

**Authors:** E. Keats Shwab, Praveen R. Juvvadi, Greg Waitt, Shareef Shaheen, John Allen, Erik J. Soderblom, Benjamin G. Bobay, Yohannes G. Asfaw, M. Arthur Moseley, William J. Steinbach

**Affiliations:** a Division of Pediatric Infectious Diseases, Department of Pediatrics, Duke University Medical Center, Durham, North Carolina, USA; b Duke Proteomics Core Facility, Institute for Genome Sciences and Policy, Duke University, Durham, North Carolina, USA; c Duke University NMR Center, Duke University Medical Center, Durham, North Carolina, USA; d Department of Biochemistry, Duke University, Durham, North Carolina, USA; e Department of Radiology, Duke University, Durham, North Carolina, USA; f Department of Laboratory Animal Resources, Duke University Medical Center, Durham, North Carolina, USA; g Department of Molecular Genetics and Microbiology, Duke University Medical Center, Durham, North Carolina, USA; University of Melbourne

**Keywords:** *Aspergillus*, filamentous fungi, fungal pathogen, protein kinase A, protein phosphorylation, proteomics

## Abstract

Protein kinase A (PKA) signaling plays a critical role in the growth and development of all eukaryotic microbes. However, few direct targets have been characterized in any organism. The fungus Aspergillus fumigatus is a leading infectious cause of death in immunocompromised patients, but the specific molecular mechanisms responsible for its pathogenesis are poorly understood. We used this important pathogen as a platform for a comprehensive and multifaceted interrogation of both the PKA-dependent whole proteome and phosphoproteome in order to elucidate the mechanisms through which PKA signaling regulates invasive microbial disease. Employing advanced quantitative whole-proteomic and phosphoproteomic approaches with two complementary phosphopeptide enrichment strategies, coupled to an independent PKA interactome analysis, we defined distinct PKA-regulated pathways and identified novel direct PKA targets contributing to pathogenesis. We discovered three previously uncharacterized virulence-associated PKA effectors, including an autophagy-related protein, Atg24; a CCAAT-binding transcriptional regulator, HapB; and a CCR4-NOT complex-associated ubiquitin ligase, Not4. Targeted mutagenesis, combined with *in vitro* kinase assays, multiple murine infection models, structural modeling, and molecular dynamics simulations, was employed to characterize the roles of these new PKA targets in growth, environmental and antimicrobial stress responses, and pathogenesis in a mammalian system. We also elucidated the molecular mechanisms of PKA regulation for these effectors by defining the functionality of phosphorylation at specific PKA target sites. We have comprehensively characterized the PKA-dependent phosphoproteome and validated PKA targets as direct regulators of infectious disease for the first time in any pathogen, providing new insights into PKA signaling and control over microbial pathogenesis.

## Abstract (precis)

PKA is essential for the virulence of eukaryotic human pathogens. Understanding PKA signaling mechanisms is therefore fundamental to deciphering pathogenesis and developing novel therapies.

## INTRODUCTION

The cAMP/protein kinase A (PKA) signaling pathway is ubiquitous and essential among all eukaryotes. Despite its critical importance for the virulence of numerous microbial pathogens, the precise mechanisms of PKA-dependent control over infectious disease remain unknown. This is mainly due to the paucity of direct PKA targets that have been well characterized in any organism. Previous studies have identified only potential PKA targets in humans and model organisms by *in vitro* proteome chip assays (1), computational prediction of target motifs (2), and targeted phosphoproteomics using enrichment with anti-PKA target motif antibodies (3).

The complexity of eukaryotic signaling pathways represents an especially daunting obstacle to defining their roles in disease caused by eukaryotic pathogens, such as invasive fungal infections. In the human-pathogenic yeast Candida albicans and the plant fungal pathogen Magnaporthe oryzae, interrogation of the PKA-dependent phosphoproteome identified potential PKA targets based on phosphorylation at canonical PKA target motifs (4, 5). However, these studies experimentally validated very few PKA targets predicted as *in vivo* substrates, and in even fewer cases has PKA-dependent site-specific phosphoregulation been investigated. Here, we comprehensively characterize the PKA phosphoproteome and validate PKA phosphorylation targets as direct regulators of microbial disease for the first time in any pathogen.

To define the roles of specific PKA signaling effectors in microbial pathogenesis, we used the fungus Aspergillus fumigatus as a prototypical eukaryotic pathogen. A. fumigatus is the primary etiology of invasive aspergillosis (IA), a leading cause of infection-associated mortality in the many immunocompromised patients treated for cancer or undergoing hematopoietic stem cell or solid-organ transplantation (6–10). Despite mortality rates of 40 to 60%, the specific molecular effectors responsible for A. fumigatus virulence are poorly understood. PKA signaling is critical for the growth and virulence of A. fumigatus (11–13), and thus, elucidation of the myriad processes regulated by the PKA signaling network will significantly advance the mechanistic understanding of overall eukaryotic microbial pathogenesis.

We employed high-resolution and quantitative liquid chromatography-tandem mass spectroscopy (LC-MS/MS) to examine the global effects of PKA on both the total fungal proteome and the phosphoproteome. Multiple proteases and two different peptide enrichment approaches, including titanium dioxide (TiO_2_) phosphopeptide enrichment and antibody enrichment of peptides containing canonical PKA target motifs, were utilized to maximize the number of unique phosphopeptides discovered. Our phosphomotif analysis, in conjunction with an independent PKA interactome analysis, identified 127 potential direct PKA targets in A. fumigatus. We characterized three probable direct PKA target proteins not previously identified as PKA effectors in any organism that represent diverse PKA-regulated pathways, including an autophagy-related protein, Atg24; a CCAAT-binding transcriptional regulator, HapB; and a CCR4-NOT complex-associated ubiquitin ligase, Not4. Furthermore, we demonstrated their impacts on disease in multiple mammalian infection systems and used a site-directed mutagenesis approach, coupled with computational modeling and molecular dynamics simulations, to define the functional effects of phosphorylation at specific PKA target sites on these effectors. This multifaceted strategy represents the most comprehensive analysis of the PKA-dependent phosphoproteome of any human pathogen, enabling the discovery of specific PKA effectors contributing to virulence. These findings have broad implications for the overall understanding of cAMP/PKA signaling mechanisms and the vital role of PKA signaling in infectious diseases.

## RESULTS

### PKA signaling impacts the abundance of proteins associated with essential cellular processes.

**FIG 1:**
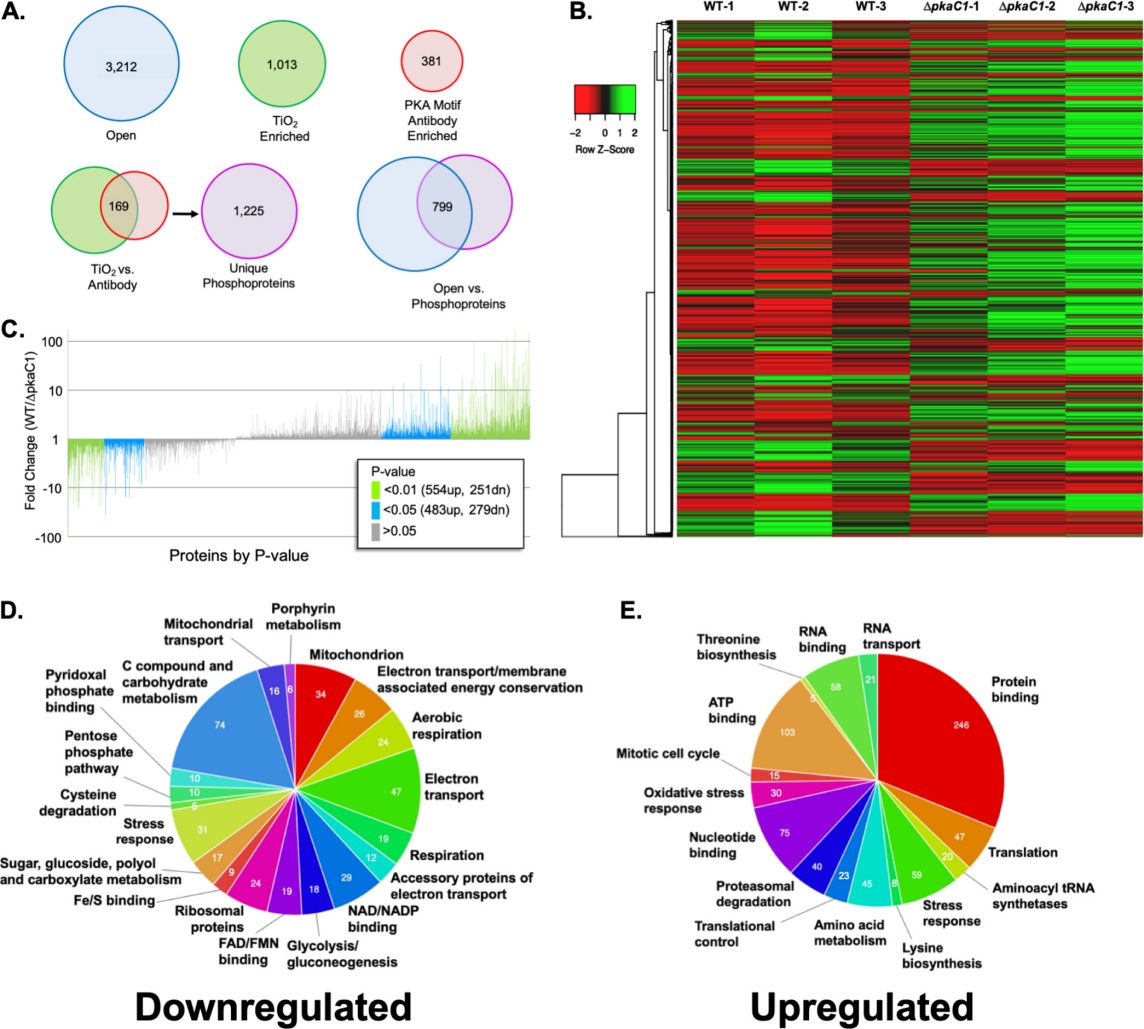
Global A. fumigatus PKA proteomic analysis. (A) Numbers of unique proteins identified in open, TiO_2_-enriched, and PKA motif-enriched data sets as well as unique phosphoproteins identified in combined TiO_2_- and PKA motif-enriched data sets. Of the 3,212 proteins identified in the open analysis, 799 were also identified in the combined phosphoprotein analyses. (B) Heat map of open protein abundances of 3,212 unique proteins identified in WT and Δ*pkaC1* samples. Red indicates a higher abundance, while green indicates a lower abundance. Brackets indicate the clustering of proteins with similar abundance profiles. (C) Fold changes in the abundances of 3,212 identified proteins in the open analysis (of 9,840 annotated A. fumigatus proteins), grouped by *P* value (increasing for negative fold changes and decreasing for positive fold changes). Proteins with statistically significant fold changes with *P* values of <0.01, *P* values of between 0.05 and 0.01, and statistically insignificant *P* values of 0.05 or higher are shown. (D) Enriched functional categories of proteins identified in the open proteomic analysis demonstrating a ≥2-fold-lower abundance in the WT genetic background than in the Δ*pkaC1* background and statistical significance of a *P* value of <0.05 in *t* test comparisons. Pie charts show enriched functional categories for this set of proteins in comparison to the total set of annotated A. fumigatus proteins based on the Functional Category (FunCat) classification system, as analyzed using FungiFun 2.2.8 bioinformatic software. FAD, flavin adenine dinucleotide; FMN, flavin mononucleotide. (E) FungiFun output of enriched functional categories of identified proteins with a ≥2-fold-higher abundance in the WT than in the Δ*pkaC1* background at a *P* value of <0.05. Pie charts show enriched categories based on FunCat classifications.

In order to determine the global effects of PKA on protein levels across the A. fumigatus whole proteome, we compared the levels from triplicate biological replicates of a wild-type (WT) A. fumigatus strain and a strain deleted for the major PKA catalytic subunit (Δ*pkaC1*) using quantitative LC-MS/MS analysis (see Fig. S1A in the supplemental material). Through this approach, 3,212 unique proteins were identified, representing approximately one-third of the estimated 9,840 proteins expressed by A. fumigatus (Fig. 1A) (14). Of these, 1,073 proteins were significantly increased in the WT strain, and 530 proteins were significantly decreased, compared to the Δ*pkaC1* strain (Fig. 1B and C; Fig. S1B). The sets of significantly upregulated or downregulated proteins were each analyzed for functional category enrichment using both the FungiFun database and the Database for Annotation, Visualization, and Integrated Discovery (DAVID). While the PKA-downregulated proteins were primarily involved in aerobic respiration (Fig. 1D; Fig. S1C and Table S1), the upregulated proteins were strongly enriched for transcriptional regulation, protein synthesis, as well as proteasomal and autophagic protein degradation in addition to numerous other functional categories (Fig. 1E; Fig. S1D and Table S2).

10.1128/mBio.02880-20.1 FIG S1 Global A. fumigatus PKA proteomic analysis. (A) Diagram of the proteomics experiment workflow. Total protein extracts from triplicate samples of A. fumigatus wild-type (WT) and Δ*pkaC1* strain mycelia were split, subjected to both LysC and GluC protease digestions, split again into three parts, and treated with either TiO_2_ for phosphopeptide enrichment or anti-PKA motif antibodies for PKA motif enrichment or unenriched for open protein analysis. LysC digests were further digested with trypsin either immediately following LysC digestion for phosphopeptide enrichment and open samples or after antibody treatment for PKA motif enrichment. (B) Volcano plot of open protein abundances of 3,212 unique proteins identified, plotted as log_2_ average fold changes in WT samples compared to the Δ*pkaC1* average (*x* axis), against the −log_10_ *P* value in a *t* test comparison of protein abundances between strain types (*y* axis). (C) Enriched functional categories of proteins identified by open proteomic analysis demonstrating a ≥2-fold-lower abundance in the WT genetic background than in the Δ*pkaC1* background and statistical significance of a *P* value of <0.05 in *t* test comparisons. Pie charts show enriched functional categories for this set of proteins in comparison to the total set of annotated A. fumigatus proteins based on the Gene Ontology (GO) and Kyoto Encyclopedia of Genes and Genomes (KEGG) classification systems, as analyzed using FungiFun 2.2.8 bioinformatic software. (D) FungiFun output of enriched functional categories of identified proteins with a ≥2-fold-higher abundance in the WT than in the Δ*pkaC1* background at a *P* value of <0.05. Pie charts show enriched categories based on GO and KEGG classifications. Download FIG S1, JPG file, 0.5 MB. Copyright © 2020 Shwab et al. 2020 Shwab et al. This content is distributed under the terms of the Creative Commons Attribution 4.0 International license.

10.1128/mBio.02880-20.7 TABLE S1 Proteins with decreased abundance in the wild type versus the Δ*pkaC1* strain in open proteomic analysis. Download Table S1, PDF file, 0.02 MB. Copyright © 2020 Shwab et al. 2020 Shwab et al. This content is distributed under the terms of the Creative Commons Attribution 4.0 International license.

10.1128/mBio.02880-20.8 TABLE S2 Proteins with increased abundance in the wild type versus the Δ*pkaC1* strain in open proteomic analysis. Download Table S2, PDF file, 0.03 MB. Copyright © 2020 Shwab et al. 2020 Shwab et al. This content is distributed under the terms of the Creative Commons Attribution 4.0 International license.

### Comparison of the PKA-dependent whole phosphoproteome and PkaC1 interactome identifies direct PKA target candidate proteins.

We compared the phosphoenriched fractions from the WT and Δ*pkaC1* strains to identify proteins that were specifically phosphorylated in a PKA-dependent manner. Enhanced coverage of phosphosites was obtained by digestion with both GluC and a combination of LysC and trypsin proteases followed by TiO_2_-based phosphopeptide enrichment (Fig. S1A). As a dual orthogonal approach, extracts were also enriched for peptides specifically phosphorylated at canonical PKA target motifs using targeted antibodies. The combination of these two data sets resulted in the identification of 1,225 uniquely phosphorylated proteins, highlighting the robustness in using the two distinct methodologies (Fig. 1A). To further identify likely direct PKA targets, we compared the abundances of each phosphopeptide between the WT and Δ*pkaC1* strains. Proteins displaying statistically significant increases in phosphopeptide abundances of at least 2-fold between the Δ*pkaC1* strain and the WT strain were prioritized (Fig. S2A). To select potential direct PKA targets from the pool of enriched phosphoproteins, the data set was further examined for phosphorylation specifically at canonical PKA target motifs, resulting in the identification of 127 unique direct PKA target candidates (Fig. S2A). These included A. fumigatus proteins previously characterized, others that are uncharacterized but homologous to proteins with known functions in other species, and also proteins with no predictable function (Table 1; Table S3).

**TABLE 1:** Functional categories of direct PKA target candidate proteins

Functional category	No. of proteins
Transcriptional regulation	48
Chromatin organization	44
Protein subcellular localization	19
Extracellular import/export	19
Cytoskeletal interaction	18
Translation	9
Lipid metabolism	9
Cellular component recycling	8
Endosomal trafficking	7
Transcription factors	7
RNA processing	6
Cell wall	3
Nuclear distribution	3
Unknown/no predicted function	38

10.1128/mBio.02880-20.2 FIG S2 Identification of direct PKA target candidate proteins. (A) Strategy for discerning likely direct PKA targets among phosphoproteins identified by LC-MS/MS analysis. The total set of identified phosphoproteins (from both TiO_2_- and antibody-enriched data sets) was narrowed to those yielding a ≥2-fold-higher abundance in the WT than in the Δ*pkaC1* genetic background with a statistical significance level of a *P* value of <0.05. This set was further narrowed to those proteins phosphorylated at canonical PKA target motifs, resulting in a set of 127 direct PKA target candidate proteins. (B) PKA target candidates were further prioritized by comparison of their PKA-dependent fold changes in phosphorylation abundance as determined by our PKA phosphoproteome analysis (*y* axis) to the strength of evidence for their interaction with the PKA catalytic subunit as estimated based on the number of coimmunoprecipitated peptide spectra identified for each protein in our PkaC1 interactome study (*x* axis). Proteins considered to be of particular interest based on a large PKA-dependent increase in phosphopeptide abundance and/or strong evidence of PKA interaction are labeled on the graph. Three proteins selected for in-depth analysis (Atg24, HapB, and Not4) are shown in color. Download FIG S2, JPG file, 0.3 MB. Copyright © 2020 Shwab et al. 2020 Shwab et al. This content is distributed under the terms of the Creative Commons Attribution 4.0 International license.

10.1128/mBio.02880-20.9 TABLE S3 Direct PKA target candidate proteins. Download Table S3, PDF file, 0.1 MB. Copyright © 2020 Shwab et al. 2020 Shwab et al. This content is distributed under the terms of the Creative Commons Attribution 4.0 International license.

To more precisely prioritize candidate PKA target proteins, the whole proteome was also interrogated by identifying proteins coimmunoprecipitated with PkaC1 in an affinity purification procedure via LC-MS/MS analysis, yielding a PKA interactome data set. As enzyme-substrate interactions tend to be transient in nature, it is likely that interactants captured in this manner may represent more frequently targeted substrate proteins. The set of 127 potential PKA targets derived from the phosphoproteome was cross-referenced against the interactome data for prioritization (Fig. S2B). In selecting PKA target candidates to pursue for in-depth characterization, three primary criteria were considered: (i) the magnitude of the fold change in phosphorylation at PKA target motifs between the WT and Δ*pkaC1* strains in the phosphoproteome data set, (ii) identification in the PKA interactome data set, and (iii) a demonstrated or predicted association with important PKA-regulated processes.

### The A. fumigatus autophagy-associated protein Atg24 is a direct PKA target *in vivo* and *in vitro*.

**FIG 2:**
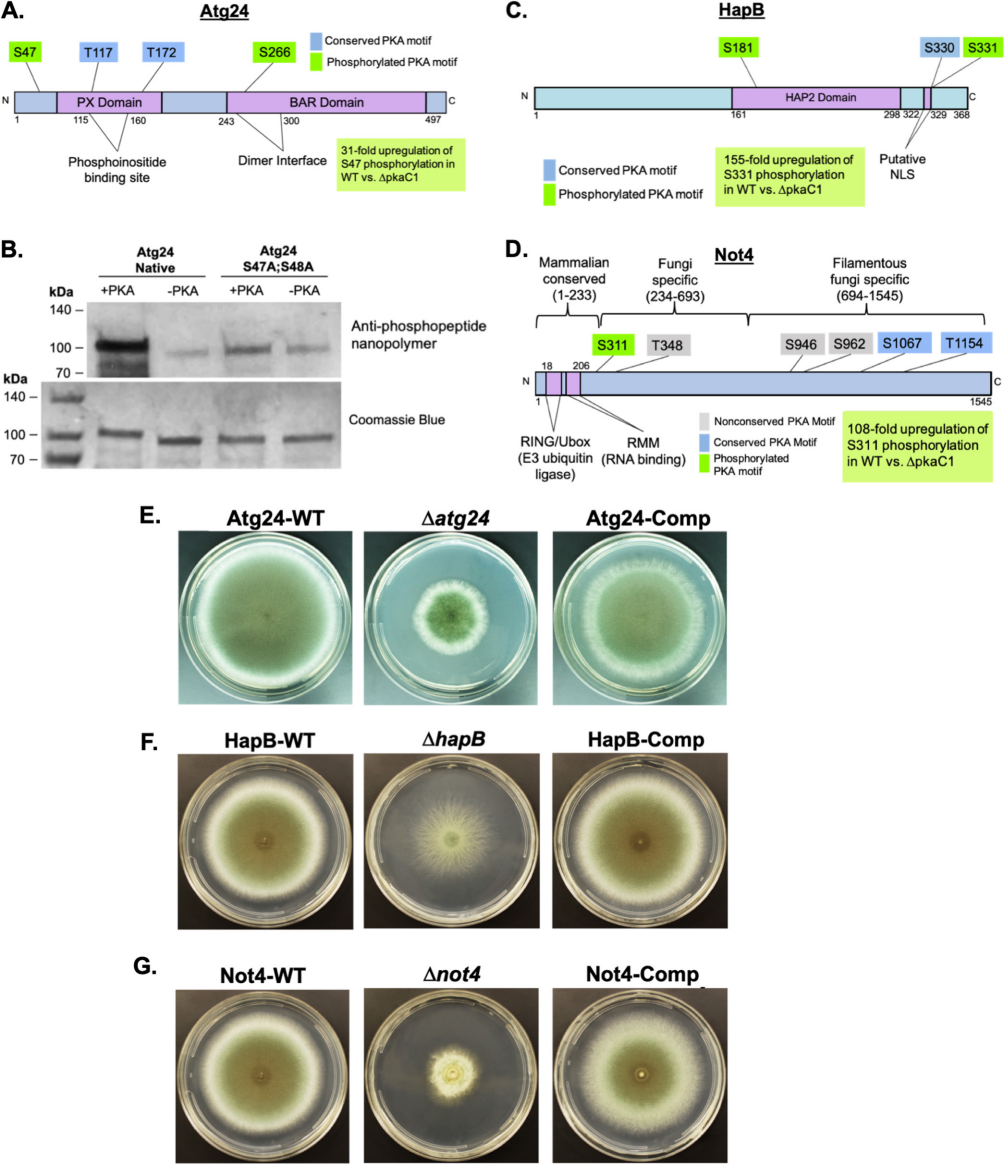
PKA-dependent phosphorylation and deletion phenotype characterization of the target proteins Atg24, HapB, and Not4. (A) Diagram of the Atg24 primary amino acid sequence indicating putative functional domains, locations of canonical PKA target motif serines (S) and threonines (T), and identified sites of phosphorylation (green boxes). (B) *In vitro* phosphorylation of recombinant Atg24 by PKA. Glutathione *S*-transferase (GST)-labeled Atg24 proteins with the native sequence and with S47 and S48 mutated to alanines were expressed in BL21 Escherichia coli cells and purified, followed by incubation either with or without the purified recombinant mammalian PKA catalytic subunit. Reaction mixture contents were separated by SDS-PAGE and either probed with antiphosphopeptide nanopolymer followed by chemiluminescent detection using X-ray film (top) or stained with Coomassie blue dye (bottom). Positions of molecular weight marker bands are indicated, and the expected size of dephosphorylated Atg24 is ∼80 kDa. An increased phosphopeptide signal was identified for native Atg24 in the presence of PKA, and a corresponding upward shift in protein migration was observed for this sample following Coomassie blue staining. (C) Diagram of the HapB primary amino acid sequence indicating the putative functional domain, locations of canonical PKA target motif serines (S), and identified sites of phosphorylation (green boxes). (D) Diagram of the Not4 primary amino acid sequence indicating putative functional domains, locations of conserved and nonconserved canonical PKA target motif serines (S) and threonines (T), the identified site of phosphorylation (green box), a region unique to filamentous fungi, a region conserved between yeast and filamentous fungi, and a region conserved between fungi and mammals. (E) Reduced-radial-growth phenotype of the Atg24 deletion strain (Δ*atg24*) grown on GMM agar for 5 days at 37°C, compared to the wild-type (Atg24-WT) and complementation (Atg24-Comp) strains. (F) Reduced-radial-growth phenotype of the HapB deletion strain (Δ*hapB*) grown on GMM agar for 4 days at 37°C, compared to the wild-type (HapB-WT) and complementation (HapB-Comp) strains. (G) Reduced-radial-growth phenotype of the Not4 deletion strain (Δ*not4*) grown on GMM agar for 4 days at 37°C, compared to the wild-type (Not4-WT) and complementation (Not4-Comp) strains.

Using the above-described stringent criteria, we identified a candidate protein (Atg24) uncharacterized among the *Aspergilli*. Atg24 is homologous to a sorting nexin essential for mitophagy, growth, conidiation, and oxidative stress tolerance in the rice blast fungus Magnaporthe oryzae (15). Interestingly, the Saccharomyces cerevisiae Atg24 homolog is involved in vacuolar protein retrieval (16), indicating potentially diverse roles for Atg24 among various eukaryotic species. A. fumigatus Atg24 was phosphorylated at two canonical PKA target motifs (Ser47 and Ser266), and phosphorylation at Ser47 was found to be 31.67-fold upregulated in the WT compared to the Δ*pkaC1* strain (Table 2 and Fig. 2A). Atg24 also contains two additional predicted PKA target motifs, but these were not identified as phosphorylated. Further evidence in support of Atg24 as a direct PKA target was provided by the identification of Atg24 in our independent PkaC1 interactome study (Table 2; Fig. S2B). The Atg24 sequence exhibits two putative conserved functional domains typical of sorting nexins: the PX domain, containing a phosphoinositide-binding region facilitating membrane association, and the BAR domain, associated with homodimerization (Fig. 2A). Both domains are essential for mitochondrial association in M. oryzae (15). As our bioinformatic analysis of the whole proteome implicated protein degradation/autophagy as a major process regulated by PKA, Atg24 represents a PKA target of potentially strong significance.

**TABLE 2:** Highlighted direct PKA target candidate proteins^*a*^

Parameter	Value	Phosphopeptide sequence	Phosphorylation site(s)	Localization probability(ies) (%)	PKA target motif	Phosphorylation fold change (WT vs Δ*pkaC1*)	Adjusted phosphorylation fold change	*P* value
Protein name	Atg24	RRM(Sp)SVHEDPPQAGPLADAVDLAGIGDGVLEC	**S47**	82.6	Yes	**25.3**	**31.67**	0.0020
GenBank accession no.	XP_751536.1	RRM(Sp)SVHEDPPQAGPLADAVDLA	S47	97.5	Yes	−1.16	1.08	0.3630
Description	Vacuolar targeting protein	RM(Sp)SVHEDPPQAGPL	S47	100	Yes	−1.53	−1.22	0.0179
No. of interactome spectra	2	RRM(Sp)SVHEDPPQAGPL	S47	99.8	Yes	−1.88	−1.50	0.0039
No. of PKA motifs	4	RE(Sp)DLEADYNDL	S266	100	Yes	−1.78	−1.42	0.0285
Expression ratio (WT/Δ*pkaC1*)	0.80	VARRE(Sp)DLEADYNDL	S266	100	Yes	−2.94	−2.35	0.0088
		RE(Sp)DLEADYNDL	S266	100	Yes	−3.5	−2.80	0.0011
		MEGDLDNDPEM(Sp)EHGR	S487	100	No	−1.64	−1.31	0.0103

Protein name	HapB	RM(Sp)HVGSPHVQ	S181	100	Yes	−1.13	1.67	0.4860
GenBank accession no.	XP_755804.1	RM(Sp)HVG(Sp)PHVQ	S181; S185	100; 100	Yes; no	1.3	2.46	0.3060
Description	CCAAT-binding transcription factor subunit	MSHVG(Sp)PHVQ	S185	100	No	1.97	3.73	0.0033
No. of interactome spectra	0	NVDSGKPAEENPSSAPKRK(Sp)SEVNDDNANSVKKS	S330	73.3	Yes	1.16	2.19	0.5290
No. of PKA motifs	3	RKS(Sp)EVNDDNANSVK	**S331**	99.2	Yes	**82.2**	**155.53**	0.0081
Expression ratio (WT/Δ*pkaC1*)	0.53	RKS(Sp)EVNDDNANSVK	S331	100	Yes	1.94	3.67	0.0345
		KS(Sp)EVNDDNANSVK	S331	99.8	Yes	−1.71	1.11	0.0029

Protein name	Not4	RA(Sp)LAGSQASQSPRPVHAT	**S311**	100	Yes	**102**	**108.84**	0.0059
GenBank accession no.	XP_750771.1	RA(Sp)LAGSQAS	**S311**	100	Yes	**20.3**	**21.66**	0.0471
Description	CCR4-NOT core complex subunit	RA(Sp)LAGSQASQSPR	**S311**	100	Yes	**15.6**	**16.65**	0.0364
No. of interactome spectra	2	RA(Sp)LAGSQ	S311	100	Yes	−2.38	−2.23	0.0094
No. of PKA motifs	6	TRRA(Sp)LAG(Sp)QASQSPRPVHAT	S311; S315	97.3; 99.5	Yes; no	1.07	1.14	0.8000
Expression ratio (WT/Δ*pkaC1*)	0.94	TRRA(Sp)LAG(Sp)QA(Sp)QSPRPVHAT	S311; S315; S318	100; 100; 99.8	Yes; no; no	4.24	4.52	0.1370
		RA(Sp)LAGSQA(Sp)QSPRPVHAT	S311; S318	100; 86.3	Yes; no	2.12	2.26	0.0325
		TAG(Tp)PPISGGGMFAQ	T638	99.7	No	1.18	1.26	0.3500
		NISEISLG(Sp)PLPK	S919	100	No	1.32	1.41	0.0879

a Lowercase “p” following parenthetically enclosed amino acid residue abbreviations within peptide sequences indicates phosphorylation of corresponding residues. Bold text indicates phosphorylated amino acid residues identified on peptide fragments with no additional phosphorylation and showing significant enrichment (*P* < 0.05) of at least 2-fold (unadjusted) in the WT strain compared to Δ*pkaC1*.

To next validate the direct phosphorylation of Atg24 by PKA, *in vitro* PKA phosphorylation assays were performed on purified recombinant Atg24 native and nonphosphorylatable alanine mutant (Atg24^S47A;S48A^) proteins. While both the native and mutant forms revealed protein bands of the expected size (∼80 kDa) in the absence of PKA, only the corresponding band for native Atg24 displayed a shift in gel migration (∼100 kDa) in the presence of PKA, indicating probable posttranslational modification at Ser47/Ser48 (Fig. 2B, bottom). Additionally, probing with an antiphosphopeptide nanopolymer confirmed the phosphorylation of WT Atg24 in the presence of PKA (Fig. 2B, top). To verify the specific site(s) of phosphorylation on Atg24, the PKA-phosphorylated native Atg24 band was analyzed via LC-MS/MS, which confirmed the phosphorylation at Ser47 with a 100% localization probability (Table S4).

10.1128/mBio.02880-20.10 TABLE S4 Atg24 *in vitro* PKA phosphorylation assay MS results. Download Table S4, PDF file, 0.02 MB. Copyright © 2020 Shwab et al. 2020 Shwab et al. This content is distributed under the terms of the Creative Commons Attribution 4.0 International license.

### The CCAAT-binding transcription factor complex component HapB is a potential direct PKA target.

Our current whole-proteomic analysis and previous studies (17, 18) indicated major roles for PKA in transcriptional control and regulation of carbon and nitrogen metabolism. To further dissect this specific aspect of PKA regulation, we characterized a second direct PKA target candidate homologous to Aspergillus nidulans HapB, a member of the CCAAT-binding transcriptional regulatory complex (CBC), not previously examined in A. fumigatus. In A. nidulans, HapB controls the nuclear localization of the CBC (19), and its deletion results in a severe growth defect with a reduced ability to metabolize several alternative carbon and nitrogen sources, including γ-aminobutyric acid (GABA) and acetamide (20). In our phosphoproteomic study, A. fumigatus HapB was identified as phosphorylated at two canonical PKA target motifs (Ser181 and Ser331) with a 100% localization probability and at a third PKA target motif site (Ser330) with a 73.3% probability. Furthermore, phosphorylation at Ser331 was found to be 155-fold upregulated in the WT compared to the Δ*pkaC1* strain when adjusted for the differential abundance of HapB in the two genetic backgrounds via open proteomic analysis (Table 2 and Fig. 2C). While the phosphorylation of Ser331 was found to be strongly PKA dependent in our phosphoproteomic analysis, a direct interaction of HapB and PKA was not identified in our PkaC1 interactome analysis (Table 2; Fig. S2B), leaving open the possibility that the phosphorylation of HapB may be regulated indirectly rather than directly by PKA. However, it is also possible that HapB was simply missed in our interactome analysis due to the transient nature of kinase-substrate interactions. The HapB sequence exhibits a conserved HAP2 domain typical of homologous CBC members. A putative nuclear localization signal (NLS) was also identified in A. fumigatus HapB based on homology to the experimentally validated NLS of A. nidulans (19) that is located immediately N terminally to the Ser330 and Ser331 phosphosites (Fig. 2C).

### The CCR4-NOT regulatory complex component Not4 is another probable direct PKA target.

Another direct PKA target candidate protein that met our stringent proteomic selection criteria was identified as Not4, which is also uncharacterized among filamentous fungi but showed sequence homology to S. cerevisiae Not4p, a member of the CCR4-NOT regulatory complex. S. cerevisiae Not4 is associated with the regulation of a number of essential cellular processes, including translation, transcription, protein degradation, and the DNA damage response (21). Not4p has also been found to be both polysome and proteasome associated, and its deletion results in increased susceptibility to translation stress (hygromycin B [HYG]), replication stress (hydroxyurea), and heat stress (22–24). While the N-terminal region of A. fumigatus Not4 shows significant homology to yeast Not4p, A. fumigatus Not4 is approximately twice the length, with its C-terminal region being absent in yeast Not4p. Interestingly, homology searches across a wide range of fungal species, including the filamentous ascomycete clade as well as ascomycete and basidiomycete yeasts and zygomycetes, revealed that the C-terminal domain is unique to filamentous fungi, suggesting a possible role in polarized growth. Moreover, the alignment of the mammalian CNOT4 protein indicated that only the extreme N-terminal region shared any significant sequence similarity between fungi and mammals (Fig. 2D; Fig. S3A). The only conserved functional domains identifiable in A. fumigatus Not4 were located in this N-terminal conserved region and included a RING/Ubox E3 ubiquitin ligase domain and an RMM RNA-binding domain (Fig. 2D). This is consistent with the role of yeast Not4p as an E3 ubiquitin ligase, known to target stalled translating peptides and the nucleosomal histone protein H2B (22, 23, 25, 26). Not4p-dependent H2B ubiquitination has also been associated with increased histone H3K4 methylation (23, 26).

10.1128/mBio.02880-20.3 FIG S3 ClustalW alignment of the amino acid sequences of Not4 homologs from human and fungal species and growth phenotypes of Not4 truncation mutants. (A) The amino acid sequences of the proteins homologous to A. fumigatus Not4 as determined by a BLAST search for Homo sapiens as well as 10 other fungal species representing the filamentous ascomycetes (A. fumigatus, A. nidulans, Penicillium brasilianum, *M. oryzae*, Neurospora crassa, and Sordaria macrospora), the ascomycete yeasts (S. cerevisiae, C. albicans, and Schizosaccharomyces pombe), as well as the basidiomycota (C. neoformans) and zygomycota (Mucor ambiguus) were aligned via the ClustalW algorithm using MegAlign v15.2.0.130 software (DNAStar). (B) Phenotypes of the Not4-WT, Not4^1–693^ amino acid truncation mutant, Not4^1–233^ truncation mutant, and Δ*not4* strains on GMM agar (5 days at 37°C). Details show an uneven leading growth edge of the Not4^1–693^ truncation mutant compared to Not4-WT. Download FIG S3, JPG file, 0.5 MB. Copyright © 2020 Shwab et al. 2020 Shwab et al. This content is distributed under the terms of the Creative Commons Attribution 4.0 International license.

Our phosphoproteomic data showed that A. fumigatus Not4 is phosphorylated at five sites with a >95% localization probability, including one canonical PKA target motif (Ser311) with a 100% localization probability. The phosphorylation of a peptide fragment at Ser311 was increased in abundance 108.84-fold in the WT background compared to the Δ*pkaC1* background (Table 2 and Fig. 2D). Additionally, two Not4 peptides were also identified in our PkaC1 interactome analysis, in addition to three other putative members of the CCR4-NOT complex (Not1, Not2, and Not3), indicating a likely interaction between the CCR4-NOT complex and PKA. Based on both phosphoproteome and interactome data and our whole-proteomic data indicating a major role for PKA in regulating protein synthesis in A. fumigatus (a primary function of CCR4-NOT in yeast), we selected Not4 as a likely direct PKA target of potentially important function.

### PKA target candidates Atg24, HapB, and Not4 are essential for hyphal growth of A. fumigatus.

To ascertain the overall importance of the probable PKA targets Atg24, HapB, and Not4 for A. fumigatus growth and development, the radial growth of deletion strains for each (Δ*atg24*, Δ*hapB*, and Δ*not4*) was compared to those of the corresponding WT strain expressing green fluorescent protein (GFP)-labeled protein (Atg24-WT, HapB-WT, and Not4-WT) and a complemented strain (Atg24-Comp, HapB-Comp, and Not4-Comp). In each case, the deletion strains exhibited markedly reduced radial growth compared to the control strains (Fig. 2E to G). To examine the impact of PKA-dependent phosphorylation at specific target residues, we also generated Atg24, HapB, and Not4 phosphomutant strains in which each of the identified phosphorylated PKA target serines and threonines (Table 2 and Fig. 2A, C, and D) was mutated to nonphosphorylatable alanine residues as well as to negatively ionizing phosphomimetic aspartic acid residues. Because A. fumigatus Not4 showed differential degrees of sequence conservation between filamentous fungi and yeasts or mammals (Fig. 2D), we also generated two mutant strains, Not4^1–693^ and Not4^1–233^, expressing truncated forms of Not4. The Not4^1–693^ mutant exhibited a slight but not statistically significant reduction in radial growth compared to Not4-WT, while Not4^1–233^ exhibited a significant radial growth defect, although this was not as severe as that of the Δ*not4* strain (Fig. S3B). A more detailed discussion of the phenotypic effects of Not4 truncation is provided in Appendix I at https://doi.org/10.6084/m9.figshare.13087418.

### Roles in stress responses and subcellular localization patterns for Atg24, HapB, and Not4.

We also identified important roles for Atg24, HapB, and Not4 in the fungal response to adverse environmental conditions, including oxidative stress, nutrient deprivation, and temperature stress, as well as to antimicrobial agents, including the three major classes of antifungals: azoles (voriconazole [VCZ]), echinocandins (caspofungin [CSP]), and polyenes (amphotericin B [AMB]), discussed in Appendix II at the URL mentioned above. Additionally, to examine the subcellular localization of the PKA target proteins Atg24, HapB, and Not4 in A. fumigatus, hyphae of the Atg24-WT, HapB-WT, and Not4-WT strains as well as selected mutant strains expressing GFP-labeled proteins were observed via live-cell fluorescence microscopy. These observations are discussed in Appendix III at the URL mentioned above.

### PKA-dependent phosphorylation of Atg24, HapB, and Not4 regulates multiple stress response pathways.

**FIG 3:**
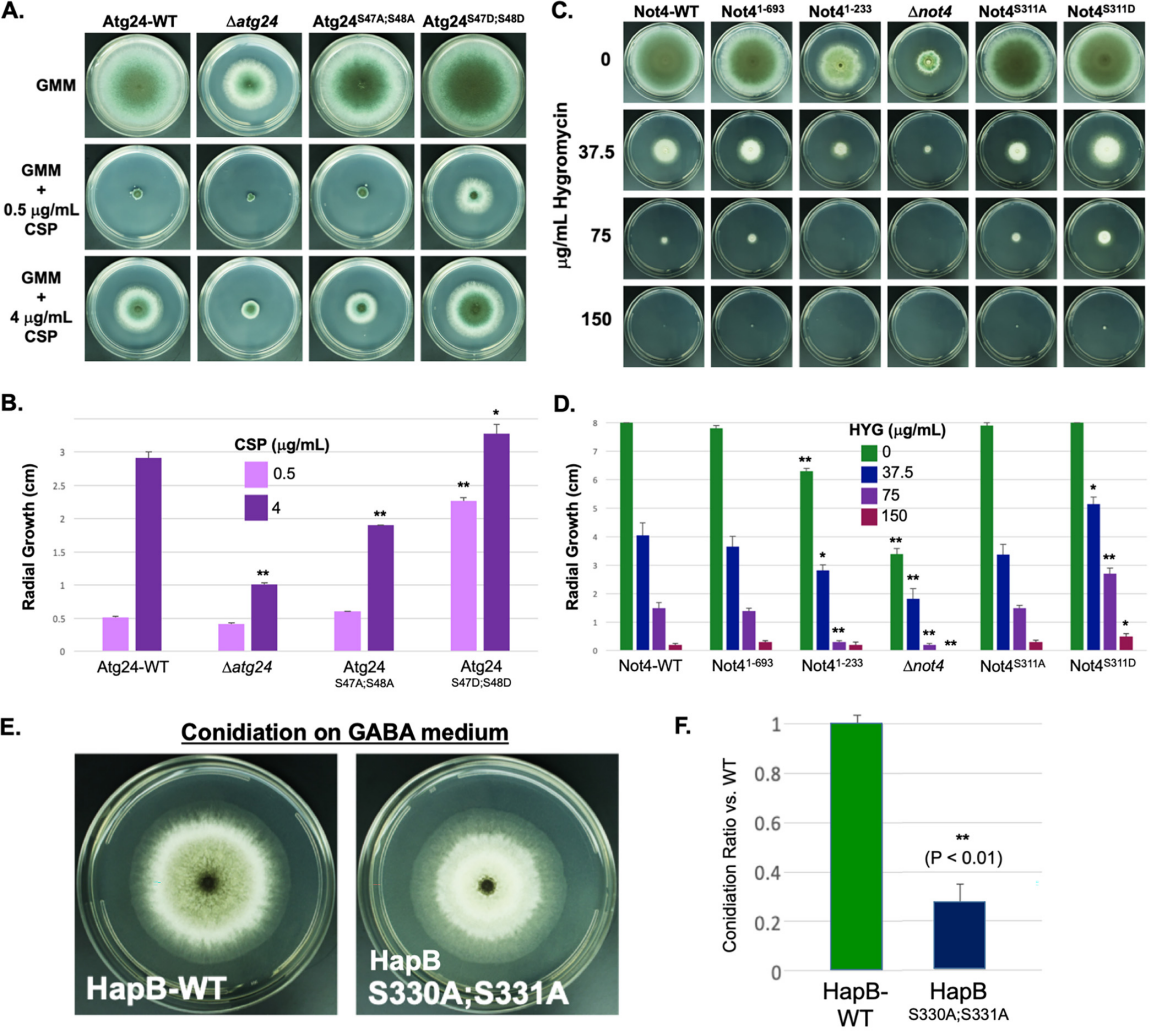
Effects of PKA-dependent phosphorylation of target proteins on stress responses. (A) Comparison of radial growth of the Atg24-WT and Δ*atg24* strains as well as Ser27/Ser48 alanine (Atg24^S47A;S48A^) and aspartic acid (Atg24^S47D;S48D^) mutant strains on GMM agar infused with 0, 0.5, or 4 μg/ml caspofungin (CSP) for 5 days at 37°C. (B) Quantitative comparison of radial growth measurements from panel A. Growth of the Δ*atg24* and Atg24^S47A;S48A^ strains was significantly more inhibited than that of Atg24-WT at 4 μg/ml CSP, while growth of the Atg24^S47D;S48D^ strain was significantly less inhibited than that of Atg24-WT at both 0.5 and 4 μg/ml CSP. (C) Comparison of radial growth of Not4-WT, Not4 truncation, Δ*not4*, and Ser311 alanine (Not4^S311A^) and aspartic acid (Not4^S311D^) substitution mutant strains on GMM agar (5 days at 37°C) infused with the indicated concentrations of hygromycin (HYG). (D) Quantitative comparison of radial growth measurements from panel C. Growth of the Not4^1–233^ and Δ*not4* strains was significantly reduced compared to Not4-WT with or without HYG (*, *P* < 0.05; **, *P* < 0.01), while Not4^S311D^ showed significantly increased growth compared to Not4-WT at all concentrations of HYG. (E) Growth (5 days at 37°C) and conidiation of HapB-WT and the HapB^S330A;S331A^ alanine substitution mutant on solid agar medium using GABA as the sole carbon and nitrogen source. (F) Quantitative comparison of conidiation on GABA medium by the HapB-WT and HapB^S330A;S331A^ strains from the experiment shown in panel E. Conidiation was significantly reduced (*P* < 0.01) in the alanine substitution mutant as determined by *t* test comparisons.

PKA deletion results in increased susceptibility to cell wall stress in A. fumigatus (12, 27), and autophagy has also been reported to play an important role in the fungal cell wall stress response (28). Therefore, we next examined whether the PKA-dependent phosphorylation of Atg24 impacts fungal cell wall stress tolerance (Fig. 3A and B). CSP, which inhibits fungal cell wall β-glucan synthesis, has been demonstrated to maximally inhibit A. fumigatus growth at lower concentrations (0.5 μg/ml), while higher concentrations (4 μg/ml) induce a “paradoxical effect” in which partial growth recovery occurs due to the activation of alternative stress response mechanisms (29). We found that the Δ*atg24* strain showed growth inhibition similar to that of Atg24-WT at 0.5 μg/ml CSP but had significantly reduced growth at 4 μg/ml. Interestingly, alanine substitution at Ser47 and Ser48 (Atg24^S47A;S48A^) also resulted in reduced paradoxical growth recovery compared to Atg24-WT at 4 μg/ml CSP despite showing growth inhibition similar to that of the control strain at 0.5 μg/ml CSP. Furthermore, the Ser47 and Ser48 phosphomimetic mutant strain (Atg24^S47D;S48D^) demonstrated markedly enhanced growth compared to Atg24-WT at 0.5 μg/ml as well as 4 μg/ml CSP. CSP tolerance was not affected by mutations at any of the other PKA target motifs. Taken together, these data indicate that the identified PKA-dependent phosphorylation of Atg24 at Ser47/Ser48 is an important regulator of the fungal cell wall stress response in A. fumigatus. However, expressing Atg24^S47D;S48D^ in a Δ*pkaC1* background failed to ameliorate the increased CSP sensitivity of the Δ*pkaC1* strain, indicating that other factors besides Atg24 phosphorylation contribute to PKA-mediated cell wall stress tolerance (Fig. S4).

10.1128/mBio.02880-20.4 FIG S4 Cell wall stress sensitivity of Atg24 and PkaC1 mutant strains. Growth of the Atg24-WT, Atg24-S47D/S48D, Δ*pkaC1*, and Δ*pkaC1*; Atg24-S47D/S48D strains was observed on agar plates infused with the indicated concentrations of caspofungin (CSP). Both the Δ*pkaC1* and Δ*pkaC1*; Atg24^S47D/S48D^ strains showed patterns of growth inhibition similar to that of Atg24-WT, with some evidence of paradoxical growth recovery at CSP concentrations of 2 and 4 μg/ml. Download FIG S4, JPG file, 0.3 MB. Copyright © 2020 Shwab et al. 2020 Shwab et al. This content is distributed under the terms of the Creative Commons Attribution 4.0 International license.

The yeast Not4 homolog has been associated with the regulation of protein translation and the response to the translation-inhibiting antibiotic hygromycin B (HYG). To determine whether PKA-dependent phosphorylation of Not4 plays a role in regulating the response of A. fumigatus to translation stress, the Not4-WT, Not4^1–694^, Not4^1–233^, Δ*not4*, Not4^S311A^, and Not4^S311D^ strains were grown on media with concentrations of HYG ranging from 0 to 150 μg/ml (Fig. 3C and D). The growth of the Not4^1–693^ and Not4^S311A^ strains was not significantly different from that of Not4-WT at any HYG concentration, while the growth of the Not4^1–233^ strain was significantly inhibited compared to Not4-WT at all HYG concentrations besides 150 μg/ml, and the Δ*not4* strain was significantly inhibited compared to Not4-WT at all HYG concentrations. Interestingly, the Not4^S311D^ mutant exhibited significantly increased growth compared to Not4-WT at 37.5, 75, and 150 μg/ml HYG, with the effect being most pronounced at 75 μg/ml. In order to further validate the contribution of Not4 to the translation stress response and determine whether these observed effects were specific to the mechanism of action of HYG, potentially through impacting glycosylation, which has been shown to contribute to HYG resistance in yeast (30), we additionally examined the growth of Not4 mutant strains under exposure to Geneticin (G418), an alternative aminoglycoside translation inhibitor (Fig. S5). While this compound was less inhibitory to A. fumigatus growth than HYG, we still found that growth reduction of the Δ*not4* strain compared to Not4-WT was exacerbated by Geneticin treatment, while the Not4^S311D^ mutant showed significantly increased growth compared to Not4-WT across a range of Geneticin concentrations. These observations suggest that Not4 plays an important role in the A. fumigatus response to translation stress and that PKA-dependent phosphorylation of Not4 at Ser311 is able to enhance the efficiency of this response. However, it remains possible that Not4 may regulate aminoglycoside tolerance through mechanisms indirectly associated with translation.

10.1128/mBio.02880-20.5 FIG S5 Effects of Not4 on Geneticin sensitivity. Quantitative comparison of the radial growth of Not4-WT, the Not4 Ser311-to-alanine (Not4^S311A^) and Ser311-to-aspartic acid (Not4^S311D^) substitution mutant strains, and the Δ*not4* strain on GMM agar (4 days at 37°C) infused with the indicated concentrations of Geneticin (G418) was performed. Statistical comparisons of each strain to Not4-WT were made at the same Geneticin concentrations (*, *P* < 0.05; **, *P* < 0.01 [by Student’s *t* test]). Growth of the Δ*not4* strain was significantly reduced compared to Not4-WT with or without Geneticin, but the ratio of Not4-WT to Δ*not4* growth was increased under Geneticin treatment (2.03 at 0 mg/ml compared to 2.85 at 3.2 mg/ml), suggesting exacerbated growth inhibition. Not4^S311D^ showed significantly increased growth compared to Not4-WT at all concentrations of geneticin examined. Download FIG S5, JPG file, 0.2 MB. Copyright © 2020 Shwab et al. 2020 Shwab et al. This content is distributed under the terms of the Creative Commons Attribution 4.0 International license.

Previously, HapB was shown to regulate alternative carbon and nitrogen source utilization in A. nidulans (19, 20). To ascertain the functional importance of HapB phosphorylation by PKA, growth of the HapB^S330A;S331A^ and HapB^S330D;S331D^ phosphomutant strains was examined on standard medium, on medium containing GABA as the sole carbon and nitrogen source, and under nitrogen starvation conditions. While the phosphosite mutation did not affect radial growth under these conditions (see Appendix II at https://doi.org/10.6084/m9.figshare.13087418), when using GABA as the sole carbon and nitrogen source, conidiation of the HapB^S330A;S331A^ strain was strongly suppressed compared to that of the HapB-WT strain (Fig. 3E and F). The ratio of Δ*hapB* to HapB-WT strain growth on GABA medium was higher than that observed for standard medium, indicating that the loss of HapB causes less severe relative growth inhibition when using GABA as the sole carbon and nitrogen source (see Appendix II at the URL mentioned above). This indicates a negative role for HapB in regulating GABA metabolism in A. fumigatus. Under such circumstances, the PKA-dependent phosphorylation of HapB at Ser330/Ser331 may have an inhibitory effect on the activity of the CBC.

### Modeling and molecular dynamics simulations of Atg24 indicate significant phosphorylation-induced electrostatic effects.

**FIG 4:**
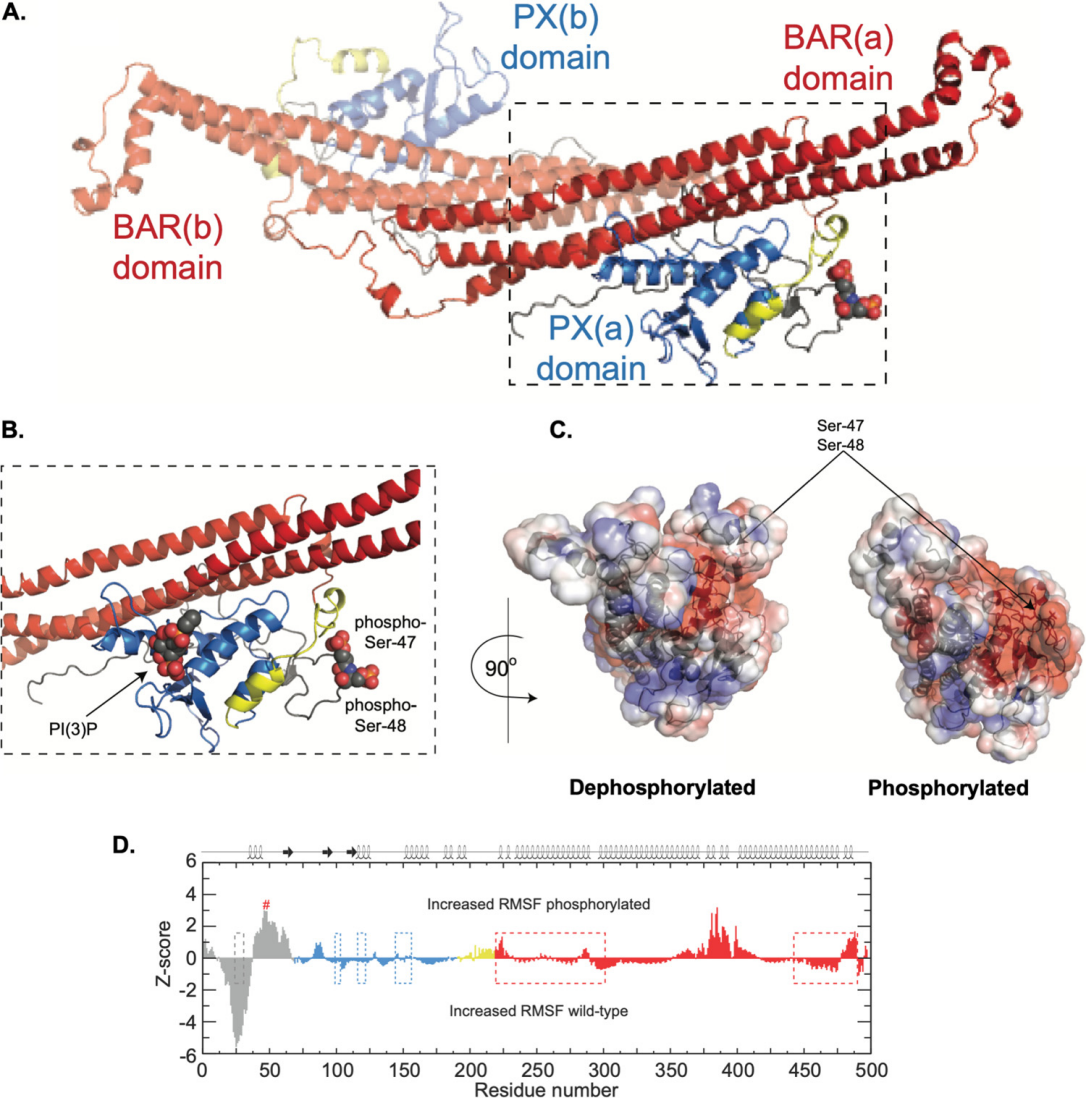
Structural models of Atg24-WT and phosphorylated Ser47 and Ser48. (A) Dimeric structure of Atg24 based on the phosphatidylinositol-3-phosphate [PI(3)P]-bound PX-BAR membrane remodeling unit of sorting nexin 9. The structure is shown in a cartoon format with the BAR domain in red, the PX domain in blue, and the sequence between the two domains in yellow. The second dimer component protein is made slightly transparent, and dimer component proteins A and B and Ser47 and Ser48, shown as spheres, are labeled for clarity. (B) Details of the site of phosphorylation of Ser47 and Ser48 as well as the PI(3)P-binding site with the same color coding as that noted above. (C) Electrostatic surface plots of the protein models created using the Adaptive Poisson-Boltzmann Solver in PyMOL for WT (left) and phosphorylated (right) proteins. The model is rotated 90° horizontally counterclockwise from the orientation shown in panel B. Blue denotes the positively charged electrostatic surface, while red denotes negatively charged areas. (D) Plot of the significance of observed root mean square fluctuation (RMSF) differences between WT and phosphorylated protein models, color coded as noted above. The secondary structure is plotted on top of the panel, dotted boxes denote residues involved in dimerization, and # denotes the site of phosphorylation for clarity. A positive Z-score suggests more significance for the phosphorylated protein, while a negative Z-score suggests more significance for the WT protein.

Because nonphosphorylatable and phosphomimetic mutations of Ser47 and Ser48 residues in Atg24 produced significant effects on protein functionality as demonstrated by the altered cell wall stress responses of these phosphomutant strains, we sought to elucidate the possible biochemical basis for this altered functionality. As homodimerization has been established as a key functional feature of BAR domain-containing proteins in general and sorting nexins in particular (31, 32), we generated *in silico* structural models of dimerized Atg24 bound to phosphoinositide-3-phosphate [PI(3)P] in both dephosphorylated and Ser47/Ser48-phosphorylated forms. The structural model of Atg24 and the subsequent dimer model (Fig. 4A) are similar to that of the PI(3)P-bound PX-BAR membrane-remodeling unit of mammalian sorting nexin 9 (33). Molecular dynamics (MD) simulations of the subsequent models revealed stability in the last 90 ns of each simulation based on the average structures from C_α_ root mean square deviations (RMSD) and radius of gyration (Rg) (a measurement of overall compactness) (Fig. S6). Phosphorylated Ser47 and Ser48 residues are located in a poorly structurally defined region of Atg24 (Fig. 4A and B, gray). The phosphorylation sites reside ∼40 Å from the PI(3)P-binding site and ∼32 Å from the nearest residues involved in dimerization. Phosphorylation of residues Ser47 and Ser48 is predicted to have several structural consequences based on the 100-ns MD simulation. Phosphorylation results in a localized change in the electrostatic surface of Atg24 (Fig. 4C); however, there is a noticeable change in the negative surface electrostatics of the localized region (expanded red region in Fig. 4C). Phosphorylation of Ser47 and Ser48 results in increases (residues 1 and 2, 39 to 56, 59 to 64, 223 to 224, 379 to 393, 398 to 400, and 483 to 486) and decreases (residues 17 to 36 and 491) in the ability of certain residues to sample conformational space (Fig. 4D, ±1 Z-score). Of these, only residues Phe223 and Val24 are also involved in dimerization. The greatest significance in RMSD differences between the WT and the phosphorylated state occurred in the N terminus (residues 1 to 68) and a loop region between alpha helices (residues 375 to 400). The MD simulation of the WT Atg24 model has three short alpha helices in its N terminus that are not observed in the MD simulation of the phosphorylated model. These findings suggest that Ser47/Ser48 phosphorylation of Atg24 is unlikely to have a dramatic impact on either dimerization or membrane association via PI(3)P binding, but due to significant localized electrostatic surface alterations, it is likely that interactions with other as-yet-undetermined binding partners could be strongly affected by this modification.

10.1128/mBio.02880-20.6 FIG S6 Visual inspection of the MD simulations of the A. fumigatus Atg24-WT and phosphorylated Ser47 and Ser48 models. The C_α_ RMSD (black and red lines) and Rg (radius of gyration) (overall compactness of structure) (green and blue lines) show stability after an initial period of equilibration (first 10 ns). Download FIG S6, JPG file, 0.3 MB. Copyright © 2020 Shwab et al. 2020 Shwab et al. This content is distributed under the terms of the Creative Commons Attribution 4.0 International license.

### Atg24, HapB, and Not4 are important virulence determinants in mammalian disease.

**FIG 5:**
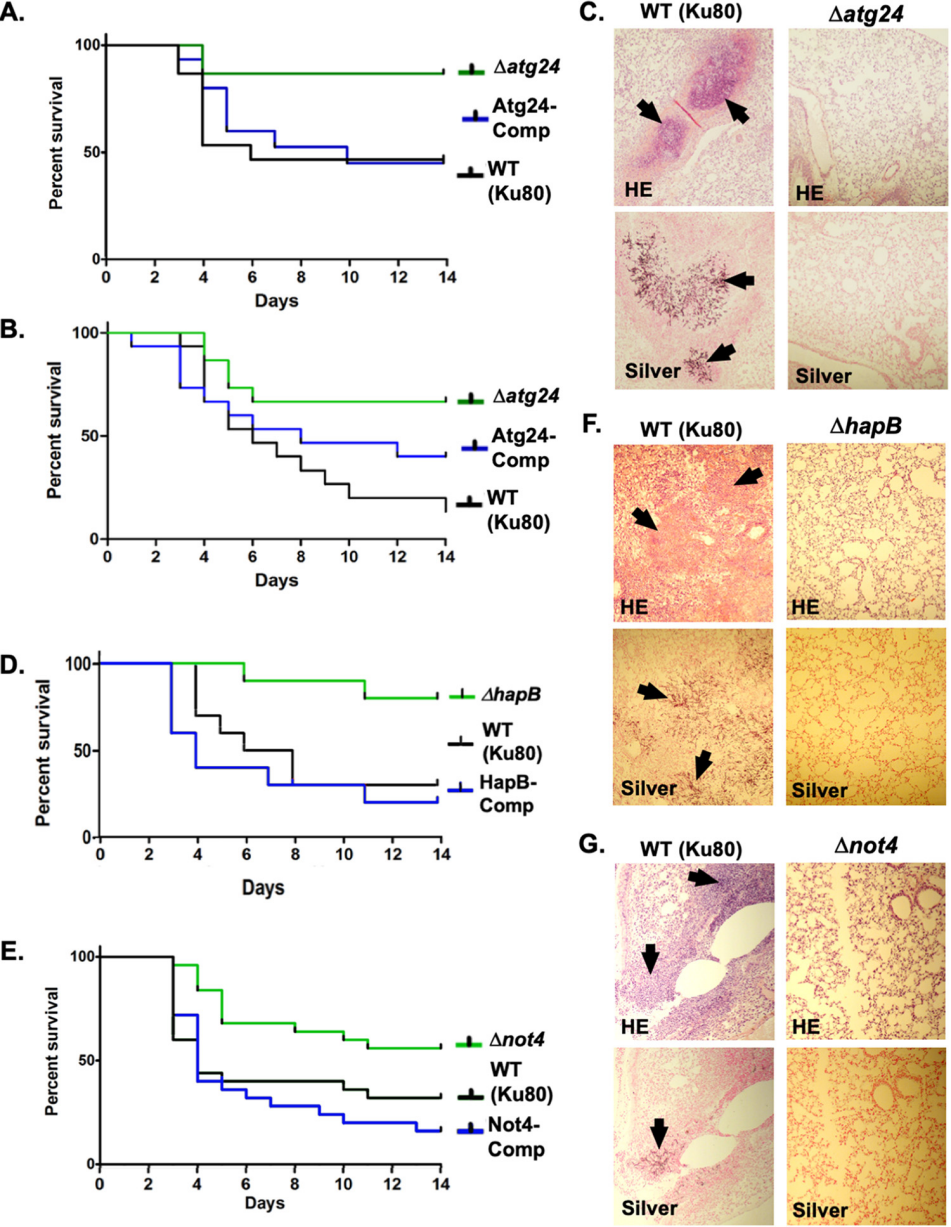
Effects of the PKA target proteins Atg24, HapB, and Not4 on virulence in a murine infection model. (A) Survival curve for Atg24 mutant strains in an inhalational murine model of invasive pulmonary aspergillosis. Fifteen mice immunosuppressed with 150 mg/kg cyclophosphamide or 40 mg/kg triamcinolone were exposed to 12 ml of aerosolized suspensions of 10^9^ spores/ml of wild-type (Ku80), Δ*atg24*, or Atg24-Comp strain conidia inside an inhalation chamber. Mice were monitored for survival for 14 days following infection. Mortality was significantly reduced for mice infected with the Δ*atg24* strain compared to the Ku80 (*P* = 0.0193) and Atg24-Comp (*P* = 0.0248) strains as determined by log rank comparisons. (B) Survival curve for repeat experiments of panel A, substituting 175 mg/kg cyclophosphamide for immunosuppression of mice. Mortality was significantly reduced (*P* = 0.0062) for mice infected with the Δ*atg24* strain compared to Ku80 but not compared to Atg24-Comp. (C) Histological analysis of mouse lung tissue 3 days after infection with Ku80 (top) or Δ*atg24* (bottom) from an experiment corresponding to the survival curve shown in panel A. Hematoxylin and eosin (HE) and silver staining were used to visualize tissue inflammation and invasive fungal growth, respectively. Examples of both are indicated by black arrows. (D) Survival curve of HapB mutant strains in the intranasal infection murine model. Ten mice immunosuppressed with 175 mg/kg cyclophosphamide or 40 mg/kg triamcinolone were inoculated with 40 μl of suspensions of 10^8^ spores/ml of Ku80, Δ*hapB*, or HapB-Comp strain conidia. Mice were then monitored for survival for 14 days following infection. Mortality was significantly reduced for mice infected with the Δ*hapB* strain compared to the Ku80 (*P* = 0.0160) and HapB-Comp (*P* = 0.0036) strains as determined by log rank comparisons. (E) Survival curve for Not4 mutant strains in the intranasal infection murine model. Twenty-five mice were inoculated as described above for panel D with Ku80,Δ*not4*, or Not4-Compstrain conidia. Mortality was significantly reduced for mice infected with the Δ*not4* strain compared to the Ku80 (*P* = 0.0295) and Not4-Comp (*P* = 0.0009) strains as determined by log rank comparisons. (F) Histological analysis of HE- and silver-stained mouse lung tissue 3 days after infection with Ku80 or Δ*hapB* conidia from the experiment shown in panel D. Arrows indicate inflammation and hyphal growth. (G) Histological analysis of HE- and silver-stained mouse lung tissue 3 days after infection with Ku80 or Δ*not4* conidia from the experiment shown in panel E. Arrows indicate inflammation and hyphal growth.

Given the strong impacts of Atg24, HapB, and Not4 on the hyphal growth and multiple stress responses of A. fumigatus, we next determined whether these PKA targets influence virulence in a mammalian host. For Atg24, immunosuppressed CD1 outbred mice were infected with conidia of the WT A. fumigatus strain (Ku80) as well as the Atg24 deletion and complementation strains via inhalation of aerosolized spores in an enclosed chamber. This experiment was repeated twice using two different levels of immunosuppression (Fig. 5A to C) for added validation. In both experiments, murine mortality was significantly reduced in the Δ*atg24* strain compared to the WT strain. In the first experiment, mortality from Δ*atg24* infection was also significantly reduced compared to Atg24-Comp, whereas in the second experiment, the difference between these strains was not statistically significant. This minor difference may be explained by the fact that the Atg24-Comp strain did not exhibit a completely recovered *in vitro* growth phenotype (Fig. S4A). Comparative histological analysis of murine lung tissues infected with the Ku80 and Δ*atg24* strains was also performed using tissue harvested at 3 days postinfection. Hematoxylin and eosin (HE) and silver staining revealed increased tissue damage and invasive hyphal growth, respectively, in the Ku80 strain-infected lung tissue compared to that infected with the Δ*atg24* strain (Fig. 5C).

To examine the contributions of HapB and Not4 to virulence, mice were infected intranasally with conidia of the Ku80, deletion, and complementation strains. Murine mortality was significantly reduced with the Δ*hapB* and Δ*not4* strains compared to both Ku80 and the respective complementation strains, while the Ku80 and complementation strains did not produce significantly different rates of mortality from one another in either experiment (Fig. 5D and E). HE and silver staining of infected lung tissue revealed increased tissue damage and invasive hyphal growth in the Ku80-infected lung tissue compared to that infected with either the Δ*hapB* or the Δ*not4* strain in each experiment (Fig. 5F and G). Overall, these results demonstrate that the activity of each of these three PKA targets contributes significantly to the disease severity of invasive aspergillosis in a mammalian host model, implicating manipulation of pathways regulated by these effectors as a potentially important mechanism for the control of pathogenesis by PKA.

### Analysis of additional direct PKA target candidate proteins indicates minor effects on fungal physiology.

In addition to Atg24, HapB, and Not4, a number of other direct PKA targets with potential significance were identified in our phosphoproteomic analysis, including both previously characterized and uncharacterized proteins. Deletion and phosphomutational analyses were carried out for several of these selected proteins (Table 3), and a detailed discussion of this work is provided in Appendix IV at https://doi.org/10.6084/m9.figshare.13087418.

**TABLE 3:** A. fumigatus strains used in this study

Strain and/or GenBank accession no.	Genotype	Reference
Ku80 (WT)	*akuB*-Ku80	62
Ku80; PyrG^−^	*akuB*-Ku80; Δ*pyrG*	62
Δ*pkaC1*	*pkaC1*::*pyrG*	27
Δ*atg24*	*atg24*::*pyrG*	This study
Atg24-Comp	*atg24*::*pyrG*; *atg24promo-atg24-hph*	This study
Atg24-WT	Δ*atg24*::*atg24promo-atg24*–*egfp-pyrG*	This study
Atg24^S47A;S48A^	Δ*atg24*::*atg24promo-atg24mt-S47A*;*S48A–egfp-pyrG*	This study
Atg24^T117A^	Δ*atg24*::*atg24promo-atg24mt-T117A–egfp-pyrG*	This study
Atg24^T172A^	Δ*atg24*::*atg24promo-atg24mt-T172A–egfp-pyrG*	This study
Atg24^T266A^	Δ*atg24*::*atg24promo-atg24mt-T266A–egfp-pyrG*	This study
Atg24^S47D;S48D^	Δ*atg24*::*atg24promo-atg24mt-S47D*;*S48D–egfp-pyrG*	This study
Δ*pkaC1*; Atg24^S47D;S48D^	*pkaC1*::*hph*; Δ*atg24*::*atg24promo-atg24mt-S47D*;*S48D–egfp-pyrG*	This study
Δ*hapB*	*hapB*::*pyrG*	This study
HapB-Comp	*hapB*::*pyrG*; *hapBpromo-hapB-hph*	This study
HapB-WT	Δ*hapB*::*hapBpromo-hapB–egfp-hph*	This study
HapB^S181A;S185A^	Δ*hapB*::*hapBpromo-hapBmut-S181A*;*S185A–egfp-hph*	This study
HapB^S330A;S331A^	Δ*hapB*::*hapBpromo-hapBmut-S330A*;*S331A–egfp-hph*	This study
HapB^S330D;S331D^	Δ*hapB*::*hapBpromo-hapBmut-S330D*;*S331D–egfp-hph*	This study
Δ*not4*	*not4*::*pyrG*	This study
Not4-Comp	*not4*::*pyrG*; *not4promo-not4trunc-1–693—egfp-hph*	This study
Not4-WT	Δ*not4*::*not4promo-not4–egfp-pyrG*	This study
Not4^1–693^	Δ*not4*::*not4promo-not4trunc-1–693—egfp-pyrG*	This study
Not4^1–233^	Δ*not4*::*not4promo-not4trunc-1–233—egfp-pyrG*	This study
Not4^S311A^	Δ*not4*::*not4promo-not4mut-S311A*; *not4trunc-1–693—egfp-hph*	This study
Not4^S311D^	Δ*not4*::*not4promo-not4mut-S311D*; *not4trunc-1–693—egfp-hph*	This study
AtfA-GFP	Δ*atfA*::*atfApromo-atfA–egfp-hph*	This study
AtfA^S234A;T379A;S433A;T441A^	Δ*atfA*::*atfApromo-atfAmut-S234A*;*T379A*;*S433A*;*T441A–egfp-hph*	This study
AmdX-GFP	Δ*amdX*::*amdXpromo-amdX–egfp-hph*	This study
Δ*pkaC1*; AmdX-GFP	*pkaC1*::*pyrG*; *ΔamdX*::*amdXpromo-amdX–egfp-hph*	This study
Rum1-GFP	Δ*rum1*::*rum1promo-rum1–egfp-hph*	This study
Δ*pkaC1*; Rum1-GFP	*pkaC1*::*pyrG*; Δ*rum1*::*rum1promo-rum1–egfp-hph*	This study
Δ*rum1*	*rum1*::*pyrG*	This study
ΔSphK (XP_752463.1)	XP_752463.1::*pyrG*	This study
ΔPst1 (XP_750917.1)	XP_750917.1::*pyrG*	This study
ΔXP_750216.1	XP_750216.1::*pyrG*	This study
ΔXP_751586.1	XP_751586.1::*pyrG*	This study
ΔXP_749288.1	XP_749288.1::*pyrG*	This study
ΔXP_001481443.1	XP_001481443.1::*pyrG*	This study

## DISCUSSION

PKA regulation is critical for cellular processes, yet its direct effectors are not well defined in any human pathogen. We identified several major PKA-regulated pathways in a pathogenic fungus and characterized for the first time key effector proteins and their specific PKA-dependent phosphoregulatory mechanisms governing disease in multiple animal models of infection. Our data support a model for PKA signaling in which dramatic changes in overall physiology and pathogenesis are regulated by finely tuned and subtle modifications to the activity of target proteins associated with myriad diverse cellular processes.

**FIG 6:**
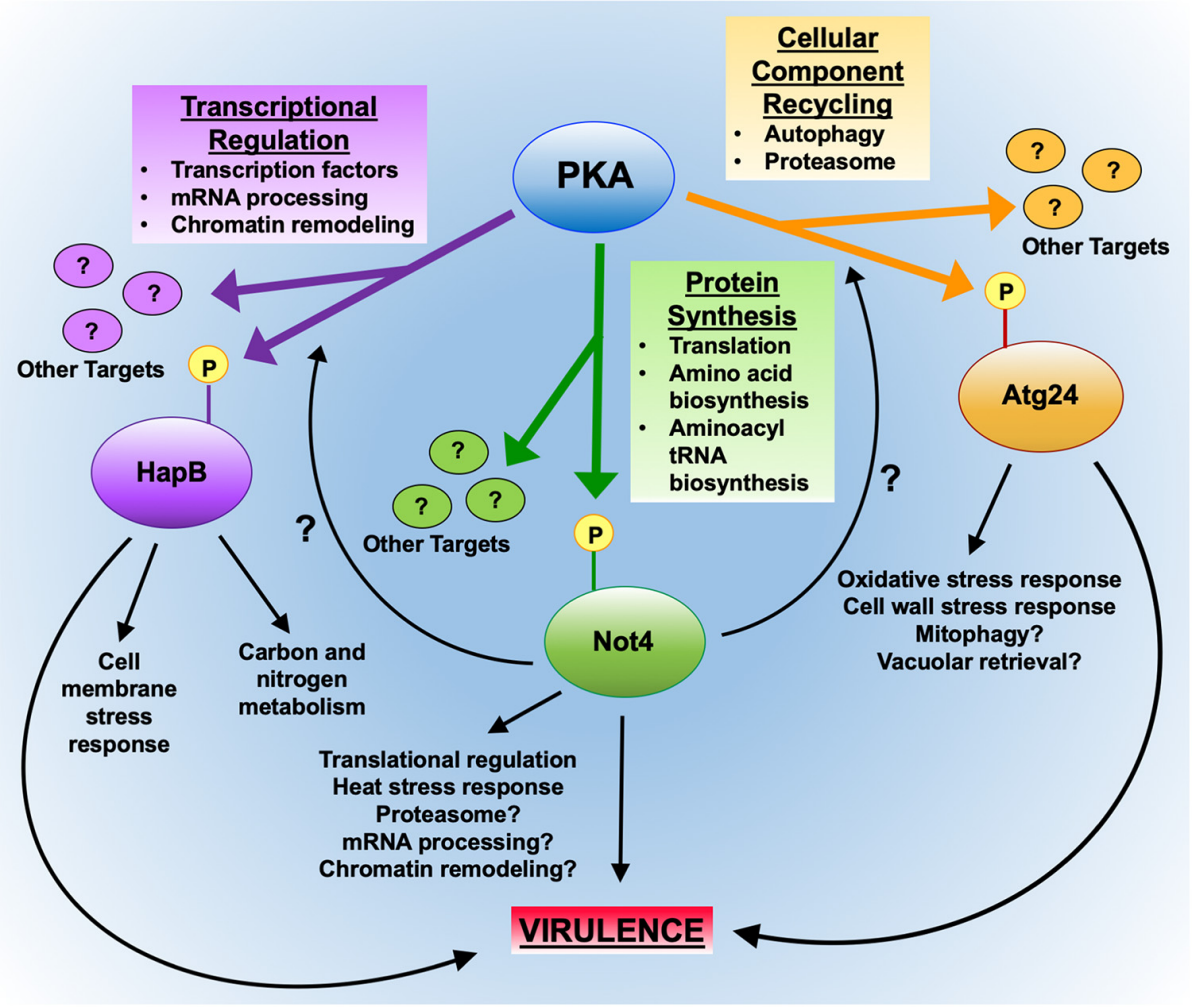
Schematic model showing new PKA signaling effectors in A. fumigatus. Major pathways regulated by PKA as evidenced by whole-proteome and phosphoproteome analyses are depicted, as are the specific roles of the PKA effectors Atg24, HapB, and Not4 in these processes. “P” indicates phosphorylation of the effector proteins by PKA. Arrows indicate regulatory pathways. Areas of regulation that are suggested but not conclusively determined by existing evidence are denoted with question marks.

The three major PKA-regulated pathways that we identified encompass fundamental cellular processes, including (i) the breakdown and recycling of cellular components via autophagy and proteasomal pathways, mediated in part through the sorting nexin Atg24; (ii) gene transcription via the regulation of chromatin remodeling and transcription factors such as the CBC component HapB; and (iii) protein biosynthesis, including amino acid and tRNA synthesis and translation, in part through the CCR4-NOT complex component Not4 (Fig. 6). Our identification of Atg24, HapB, and Not4 each as both probable direct PKA targets and important contributors to pathogenesis in a mammalian host demonstrates for the first time a direct link between PKA signaling and the respective pathways associated with each of these effectors in the virulence of a human pathogen. Importantly, neither Atg24 nor Not4 homologs have previously been associated with virulence in any pathogen. Another CBC component, HapC, was previously shown to be essential for the virulence of A. fumigatus (34), which together with our identification of HapB as an important virulence determinant strongly supports a role for the CBC in A. fumigatus pathogenesis. While an association with virulence has been previously reported for autophagy-associated proteins (35–37), CBC components (38), and CCR4-NOT complex members (39–41) in some pathogenic yeast species, our findings suggest a key role for PKA in regulating the impacts of these processes on pathogenesis. Considering the significant but relatively modest functional effects observed from individual phosphosite mutations in the PKA effectors, it is unlikely that the phosphorylation of any single target protein alone would have a major influence on pathogenesis. Instead, our results suggest that the overall dramatic impact of PKA signaling on fungal virulence is a result of the cumulative effects of regulatory modification of numerous effector proteins involved in diverse cellular pathways. Given the divergence of these pathways between mammals and microbial pathogens, elucidating their poorly defined mechanisms to identify novel pathogen-specific processes may lead to new targeted therapeutic strategies.

Such mechanistic insights were possible through a unique combination of robust quantitative whole-proteomic and phosphoproteomic approaches using dual-phosphopeptide enrichment strategies with three different proteases to maximize proteome coverage. Quantitative comparison between triplicate samples of each strain confirmed the identification of phosphorylated proteins statistically significantly enhanced exclusively by PKA activity. This quantitative approach is beneficial because many proteins may be phosphorylated by multiple kinases, even at the same target sites, and such proteins might thus be phosphorylated to some degree even in the absence of one targeting kinase and therefore overlooked in a qualitative screen. Integration of the PKA-dependent phosphoproteome data with PkaC1 interactome data enabled the most thorough interrogation of direct PKA target proteins that has been performed for any organism.

Of the 3,212 proteins quantified through open proteomic analysis, the abundances of approximately half were significantly altered by PKA, confirming its role as a master regulator of biological processes. Of the proteins upregulated by PKA, tandem bioinformatic approaches revealed that those involved in both protein expression and degradation were prominent, indicating that a primary mode of action for PKA is in the modulation of protein levels. This observation that PKA regulates protein homeostasis is in accordance with the PKA-dependent phosphoproteome of Cryptococcus neoformans, in which proteasomal regulation by PKA was found to influence capsule formation, and recent work in C. albicans, wherein proteasomal inhibition induced filamentation in a PKA-dependent manner (42, 43). The molecular chaperone Hsp90 also negatively regulates filamentation in C. albicans through suppression of PKA signaling (44), suggesting a major role for PKA in filamentous fungal growth, which may have implications for polarized cell growth among eukaryotes. We found that the most prominent functional category downregulated by PKA was proteins involved in aerobic respiration, illustrating the role of PKA as a regulatory check on the metabolic rate. The majority of the 127 strongly predicted PKA target proteins serve putative functions in transcriptional regulation, either as DNA-binding transcription factors or through chromatin remodeling, indicating this as a primary direct method by which PKA exerts its influence over cellular processes.

We validated the putative autophagy-associated protein Atg24 as an *in vitro* and *in vivo* target of PKA and demonstrated the functional significance of the PKA-dependent phosphorylation of Atg24. PKA has been previously linked to autophagic processes in S. cerevisiae, targeting the autophagy-associated proteins Atg1p and Atg13p (45, 46). In the plant pathogen *M. oryzae*, Atg24 may function similarly to yeast Atg32p, an essential component of the yeast mitophagy pathway (15, 47). A. fumigatus Atg24 also exhibits sequence similarity to the yeast sorting nexin Snx4p, involved in protein retrieval from vacuoles (16), and it is possible that A. fumigatus Atg24 is involved in either or both of these processes.

Molecular modeling and MD simulations of the nonphosphorylated and phosphorylated states of Atg24 provided a biochemical basis for the functional consequences of Atg24 phosphorylation by PKA, indicating that the phosphorylation of Ser47/Ser48 drastically altered the negative electrostatic character of the protein albeit in a localized manner. The Atg24 mammalian ortholog sorting nexin 9 contains an N-terminal amphipathic helix important for membrane tubulation/anchoring (33), and our MD simulation revealed a loss of three short alpha helices in the N terminus upon phosphorylation. This suggests that the phosphorylated state may still dimerize and bind PI(3)P; however, it may not associate efficiently with target membranes.

The effects of Atg24 PKA phosphosite mutations on the response to the antifungal caspofungin are of broad clinical relevance given the treatment resistance challenges of invasive fungal infections (48–51). Although an association between autophagy and the cell wall stress response has been reported (28), mechanisms by which the processes are related remain unclear. One possibility is that autophagy facilitates the fungal response to cell wall stress by freeing up resources from nonessential cellular components that may instead be redirected toward cell wall repair mechanisms. Our findings should facilitate investigation into new methods for enhancing the efficacy of cell wall-targeting antifungal agents.

We also present novel evidence of a specific mechanism for nutrient sensing regulation through the PKA-dependent phosphorylation of the CBC component HapB, the first demonstration of CBC regulation by PKA. The CBC is conserved among eukaryotes and has been found to regulate growth on nonfermentable carbon sources in S. cerevisiae (52) and to regulate both iron and redox homeostasis in A. nidulans (53, 54). It was also shown to regulate the expression of the acetamidase-encoding *amdS* gene of A. nidulans (55). Another transcription factor known to promote *amdS* expression in A. nidulans, AmdX (56), was also identified in our study as a direct PKA target, suggesting the possibility of coordinated regulation of metabolic pathways by PKA through multiple avenues. Additionally, the markedly reduced conidiation resulting from HapB phosphosite mutation during growth using GABA as the sole carbon and nitrogen source indicates an important role for PKA in modifying HapB function. Although HapB phosphosite mutation did not influence the nuclear localization of the protein, PKA-dependent phosphorylation of HapB may affect its association with other components of the CBC.

This work also provides the first identification of the E3 ubiquitin ligase CCR4-NOT complex component Not4 as a probable direct PKA target in any organism. A functional association between PKA signaling and CCR4-NOT activity has been reported in that both pathways appear to coordinate the repression of the stress response mediated by the Msn2/4p transcription factors in yeast (57). A PKA target site of Not4 regulating tolerance to HYG suggests an important role for PKA in regulating CCR4-NOT translational control. In addition, the identification of Not1, Not2, Not3, and Not4 in our PkaC1 interactome as well as a recent report on the identification of PKA-dependent Not3 phosphorylation in A. nidulans (58) together suggest a high degree of regulatory interaction between PKA and the CCR4-NOT complex. The identification of a filamentous fungus-specific C-terminal domain of Not4 is novel, as no component of the CCR4-NOT complex has previously been characterized in a filamentous fungus. Our observation of the dramatically increased abundance of truncated Not4 proteins (see Appendix III at https://doi.org/10.6084/m9.figshare.13087418) suggests a potential role for this C-terminal domain in regulating the expression and/or degradation of Not4. Additionally, the subcellular localization of the Not4 truncations indicates that the domain may be involved in nuclear import/export mechanisms.

These data comprise the most comprehensive analysis of direct and validated PKA targets in any organism and provide new insights into the specific ways in which PKA executes its role as a master regulator of biological signaling pathways governing overall microbial pathogenesis. Understanding how this ubiquitous signaling pathway controls virulence in all pathogens will allow the future development of targeted therapies for disease.

## MATERIALS AND METHODS

### Protein extraction and purification.

A. fumigatus strains were cultured in liquid glucose minimal medium (GMM) with shaking at 250 rpm for 24 h at 37°C. The total cell lysate was obtained by homogenizing mycelia (1 g [wet weight]) using a mortar and pestle as previously described (27, 59, 60). The total protein in the crude extracts was quantified by the Bradford method (61), and samples were normalized to contain 10 mg of protein each. GFP-Trap (ChromoTek, Planegg-Martinsried, Germany) affinity purifications were performed from crude extracts according to the manufacturer’s instructions, as previously described (59).

### Quantitative LC-MS/MS analysis.

Triplicate liquid GMM cultures of WT and Δ*pkaC1* strains were grown for 24 h at 37°C. The whole protein from each sample was extracted via bead homogenization with 8 M urea. Either LysC or GluC was added, and growth was allowed to proceed for 18 h. After digestion, the protein was subjected to antibody enrichment, trypsin digestion, and, finally, TiO_2_ enrichment, as applicable. Phosphopeptide enrichments were performed using TiO_2_ spin tips (GL Bioscience). For anti-PKA phosphopeptide antibody enrichment, resuspended peptides were then transferred in immunoaffinity purification buffer directly onto prealiquoted phospho-PKA substrate beads (Cell Signaling Technology). Immunoprecipitation was performed for 2 h at 4°C. Combined eluents were further enriched using TiO_2_ spin tips (GL Bioscience). Quantitative LC-MS/MS was performed using a nanoAcquity ultraperformance liquid chromatography (UPLC) system (Waters Corp.) coupled to a Thermo Fusion Lumos high-resolution accurate-mass tandem mass spectrometer via a nanoelectrospray ionization source. Analytical separation was performed using a 1.7-μm Acquity BEH130 C_18_ 75-μm by 250-mm column (Waters Corp.) with a 90-min linear gradient of 3% to 30% acetonitrile (for open analysis, 5 to 30% acetonitrile). Data collection on the Fusion Lumos mass spectrometer was performed in the data-dependent acquisition (DDA) mode of acquisition. Following 9 total UPLC-MS/MS analyses for each open analysis set, data were imported into Proteome Discoverer 2.2 (Thermo Scientific Inc.). The relative peptide abundance was calculated based on the areas under the curve of the selected ion chromatograms. The MS/MS data were searched against the NCBI Aspergillus fumigatus database. Mascot Distiller and Mascot Server (v2.5; MatrixSciences) were utilized to produce fragment ion spectra and perform the database searches. To identify differences between the WT and Δ*pkaC1* groups, a two-tailed *t* test was performed on log_2_ protein or phosphopeptide intensities. Fold changes were calculated by determining ratios of the average intensities. Considering something differentially expressed between the groups required a *P* value of <0.05 and a fold change of ≥2.

### Bioinformatic analysis of protein functional cluster enrichment.

Sets of significantly upregulated or downregulated proteins from the open proteomic analysis were each analyzed for the enrichment of particular functional categories using both the FungiFun v2.2.8 (https://elbe.hki-jena.de/fungifun/) and DAVID v6.8 (https://david.ncifcrf.gov/) bioinformatic programs. Both programs compared sets of significantly upregulated or downregulated proteins to the complete set of annotated A. fumigatus (Af293) proteins to identify disproportionate enrichments of proteins associated with particular functions based on multiple categorization schemes.

### Construction of gene deletions and mutations in A. fumigatus.

Genotypes of the strains used in this study are detailed in Table 3 (27, 62). Mutant strains were generated as described previously (27). Briefly, GFP labeling of proteins was accomplished by the insertion of the appropriate coding region 5′ to, and in frame with, the *egfp* coding region in a plasmid vector, followed by transformation of the *akuB*^KU80^ strain of A. fumigatus and screening via HYG or *pyrG* selection as described previously (60). Gene deletions were accomplished via the replacement of the open reading frame of interest with the Aspergillus parasiticus *pyrG* selectable marker. Deletions were verified via PCR and Southern blotting as described previously (63). Plasmid constructs for site-directed mutagenesis and gene truncations were generated via fusion PCR as described previously (27). All mutated strains were verified via DNA sequencing. The presence of GFP-labeled mutant protein forms was verified for key strains by Western blotting with anti-GFP antibodies as described previously (60). Phosphorylation of purified Escherichia coli-expressed recombinant Atg24 protein was detected via western blot probed with pIMAGO biotin-conjugated anti-phosphopeptide nanopolymers (Tymora Analytical Operations, West Lafayette, Indiana) followed by detection with avidin-conjugated horseradish peroxidase and chemiluminescent substrate according to manufacturer's instructions.

### Assessment of radial growth, conidiation, and antifungal compound susceptibility.

For radial growth assays, conidia (10^4^) were point inoculated onto GMM agar medium in petri plates and incubated for 4 or 5 days at 37°C. Growth media used included standard GMM (1% glucose, pH 6.5) as well as modified GMM containing either 50 mM H_2_O_2_, 0.5 μg/ml CSP, 4 μg/ml CSP, 3.75 μg/ml HYG, 75 μg/ml HYG, or 150 μg/ml HYG. Conidiation was quantified by collecting conidia from each plate in equal volumes of 0.05% Tween 20, followed by dilution of the collected spore stocks and counting of spores via a hemocytometer. The mean radial growth and conidiation rates for each of the strains were compared statistically by Student’s *t* test. For CSP, VCZ, and AMB sensitivity determinations, CLSI M38-A2 *in vitro* antifungal susceptibility standards were used (64).

### Modeling and molecular dynamic simulations.

A structural model of monomeric Atg24 was created using iTASSER (65–67). The created structural model was then used to model the phosphorylated (Ser47 and Ser48) protein created through PyMOL (PyMOL Molecular Graphics System version 1.2r3pre; Schrödinger LLC). Molecular dynamics (MD) simulations of structural models were performed with the GROMACS 5.1 software package using the CHARMM force field and the flexible TIP3 water model individually (68). The initial structures were immersed in a periodic water box with a dodecahedron shape (1-nm thickness) and neutralized with counterions. The electrostatic energy was calculated using the particle mesh Ewald method with 0.9-nm cutoff distances for the Coulomb and van der Waals interactions. After energy minimization, the system was equilibrated to 300 K and normal pressure for 100 ps with position restraints for heavy atoms and Linear Constraint Solver (LINCS) constraints for all bonds. The system was coupled to the external bath by Parrinello-Rahman pressure and temperature coupling. The final MD calculations were performed under the same conditions except that the position restraints were removed and the simulation was run for 100 ns. The last frame of the 100-ns simulation was extracted for each model for electrostatic surface analysis. PyMOL Molecular Graphics System version 1.8 (Schrödinger) was used for structural analysis and image creation. The electrostatic surface plots of the protein models were created by using the Adaptive Poisson-Boltzmann Solver in PyMOL (69, 70). The PDB2PQR Web server (71) was used with the CHARMM force field and output naming schemes with a default pH of 7.5.

### Murine invasive aspergillosis virulence assays and histopathological analysis.

Mice weighing 13 to 16 g (CD1; Charles River Laboratory, Raleigh, NC, USA) were immunosuppressed with cyclophosphamide (150 mg/kg of body weight for intranasal infection or 175 mg/kg for inhalational infection, intraperitoneally, on days −2 before infection and +3 postinfection) and triamcinolone acetonide (40 mg/kg, subcutaneously, on days −1 and +6). For intranasal infections, 40 μl of suspensions of 1 × 10^8^ conidia/ml of the relevant strains was delivered intranasally following brief isoflurane anesthesia induction. For inhalational infections, mice were placed in an enclosed plexiglass chamber aerosolized with 12 ml of a spore suspension containing 10^9^ conidia/ml for 1 h. Survival was plotted on a Kaplan-Meier curve and analyzed using log rank pairwise comparisons. *P* values of <0.05 were considered statistically significant. To characterize disease histopathology, additional mice were infected with each of the analyzed strains. Mice were euthanized on day +3 after infection, and lungs were harvested. Lung sections were stained with Gomori’s methenamine silver stain to visualize fungal hyphae and with hematoxylin and eosin stain to examine inflammation and tissue damage, as previously described (72). Animal studies at the Duke University Medical Center were performed in full compliance with all of the guidelines of the Duke University Medical Center Institutional Animal Care and Use Committee (IACUC) and in full compliance with the U.S. Animal Welfare Act (Public Law 98-198). The Duke University Medical Center IACUC approved all of the vertebrate studies under protocol number A-249-16-11. The studies were conducted in the Division of Laboratory Animal Resources (DLAR) facilities, which are accredited by the Association for Assessment and Accreditation of Laboratory Animal Care (AAALAC).

### Fluorescence microscopy.

Conidia (10^3^) of the respective GFP-labeled strains were cultured in 35-mm cover-glass-bottom petri dishes containing 3 ml of liquid GMM and incubated for 16 h at 37°C prior to visualization. For mitochondrial staining, where noted, 3 μl of MitoTracker dye (Invitrogen) was added to the growth medium 15 min prior to visualization. Hyphae were visualized using an Axio Observer 3 microscope (Carl Zeiss, Oberkochen, Germany) equipped with ZEN Lite imaging software.

### Data availability

Raw phosphoproteome data are accessible at the Duke Center for Genomic and Computational Biology Express Data Repository: https://discovery.genome.duke.edu/express/resources/5081/.
