# Supplementary figures and images for: The Protein Kinase A-Dependent Phosphoproteome of the Human Pathogen Aspergillus fumigatus Reveals Diverse Virulence-Associated Kinase Targets
Source: mBio. 2020 Dec 15;11(6):e02880-20. doi: 10.1128/mBio.02880-20 (PMC7773993; doi:10.1128/mBio.02880-20)

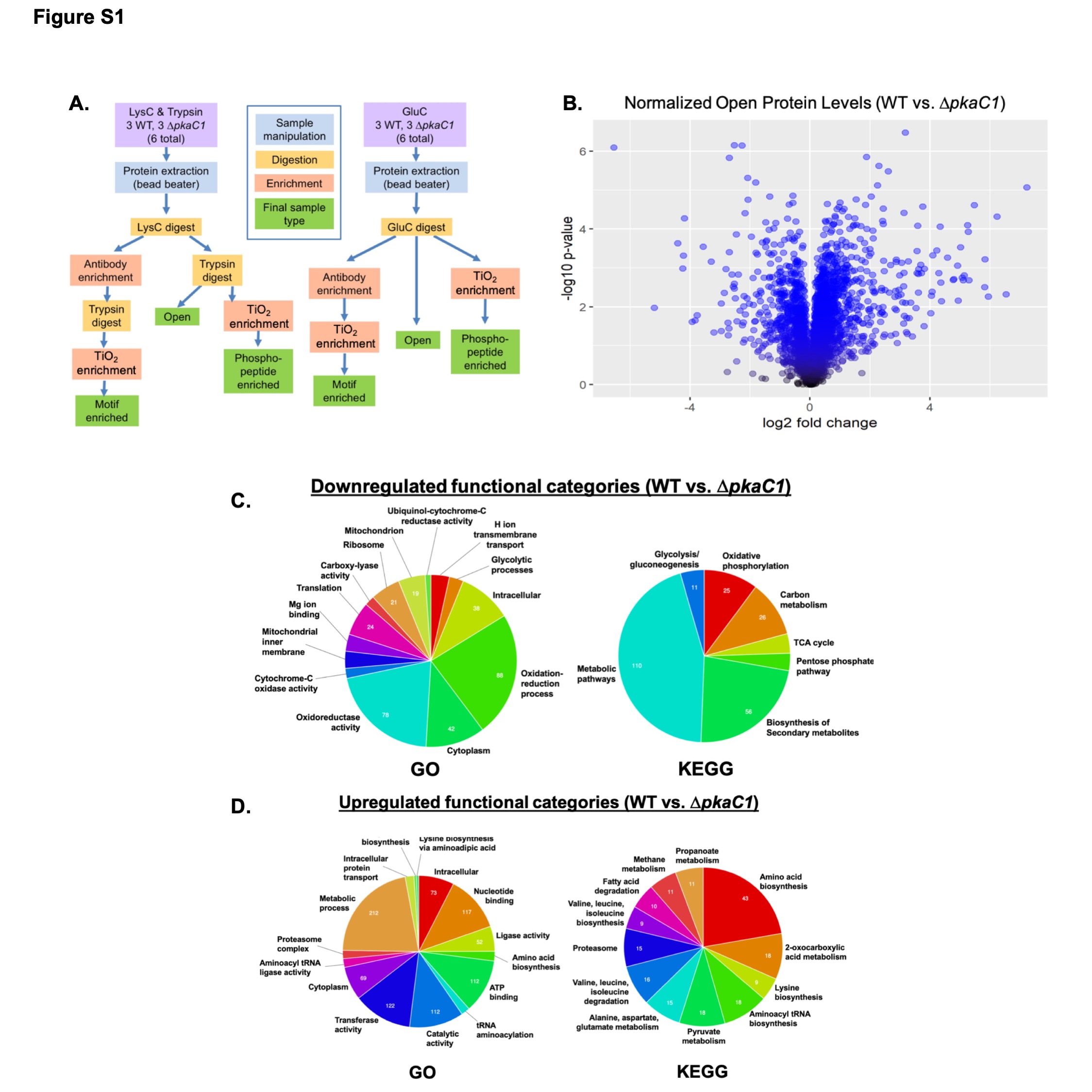

Supplement: FIG S1 [file mBio.02880-20-sf001.jpg]

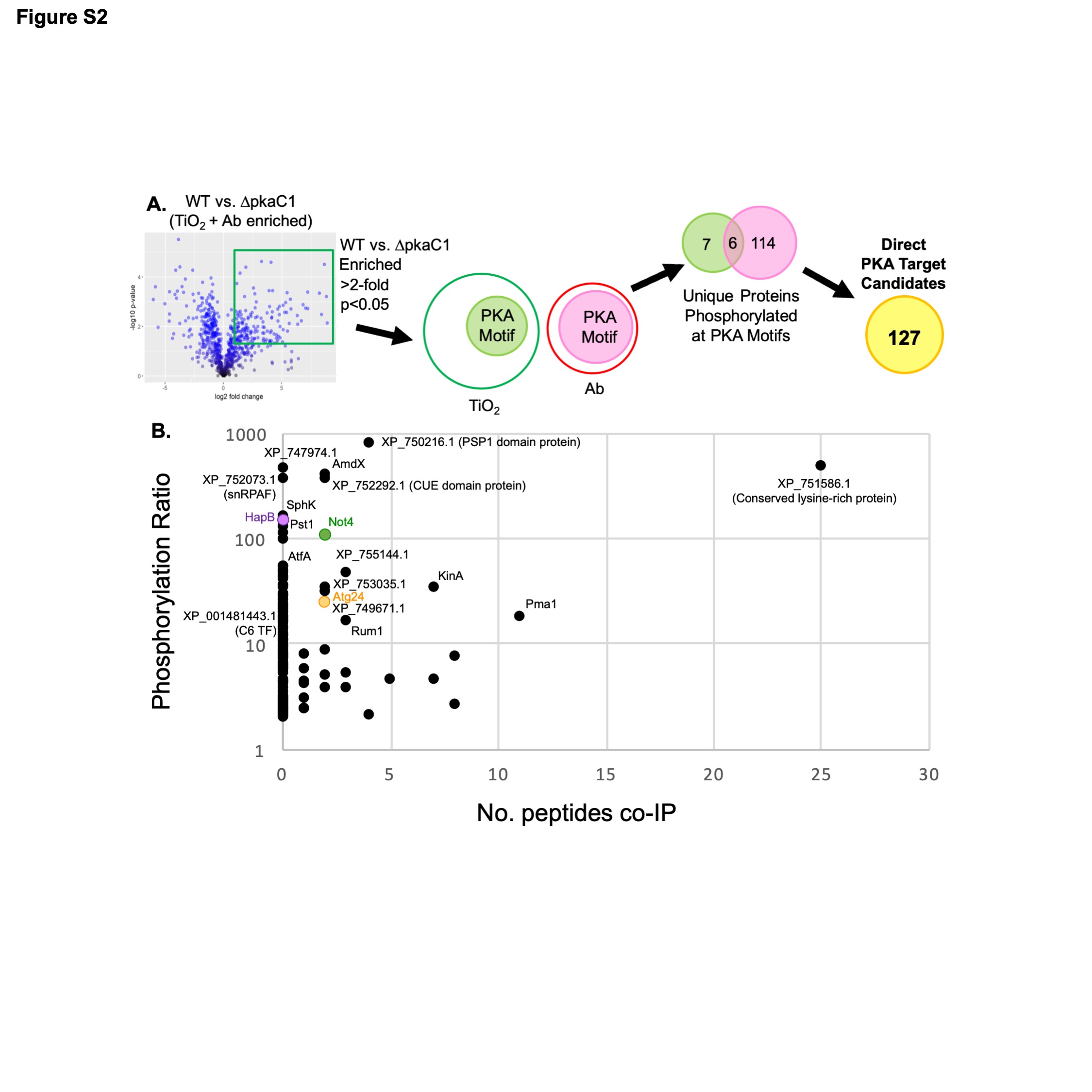

Supplement: FIG S2 [file mBio.02880-20-sf002.jpg]

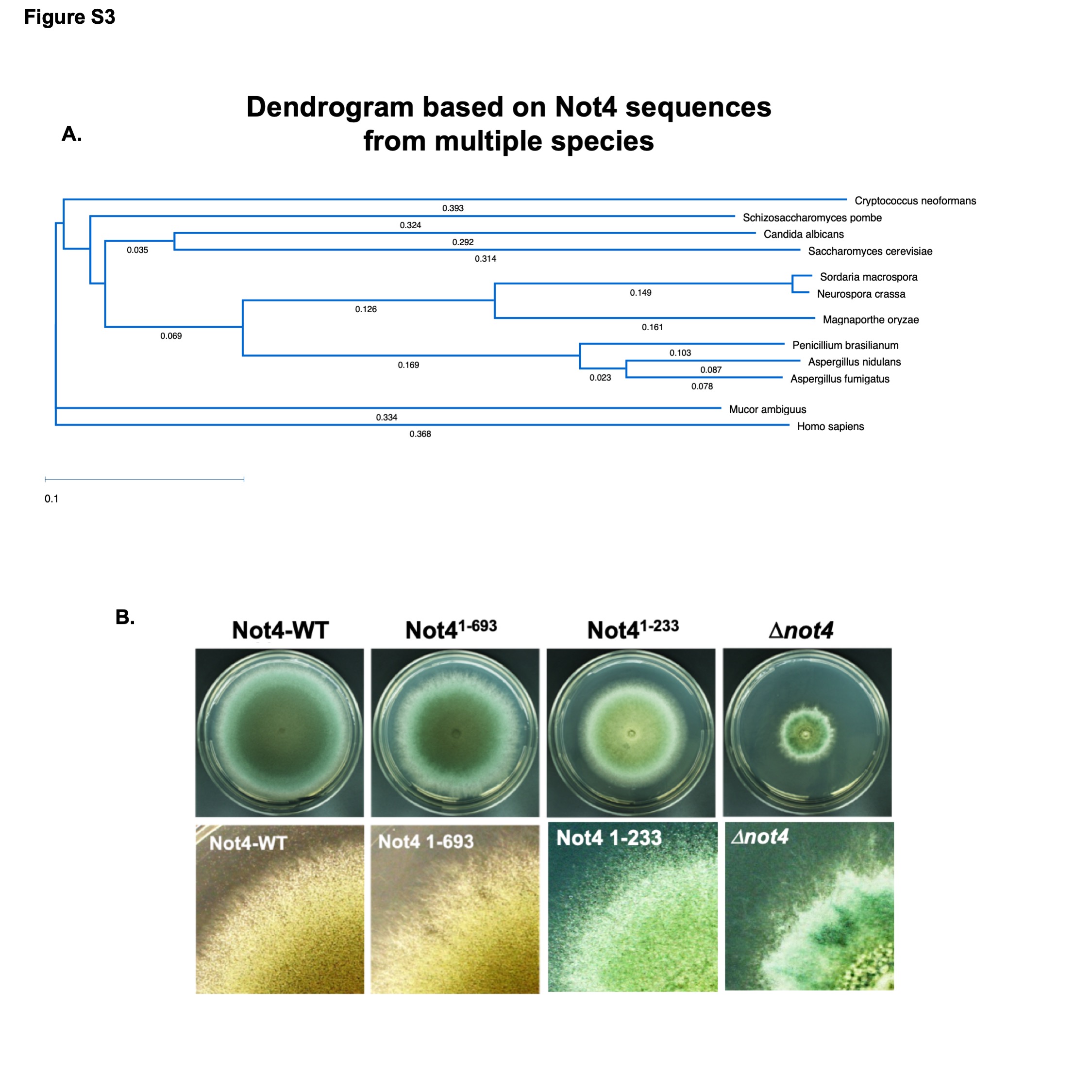

Supplement: FIG S3 [file mBio.02880-20-sf003.jpg]

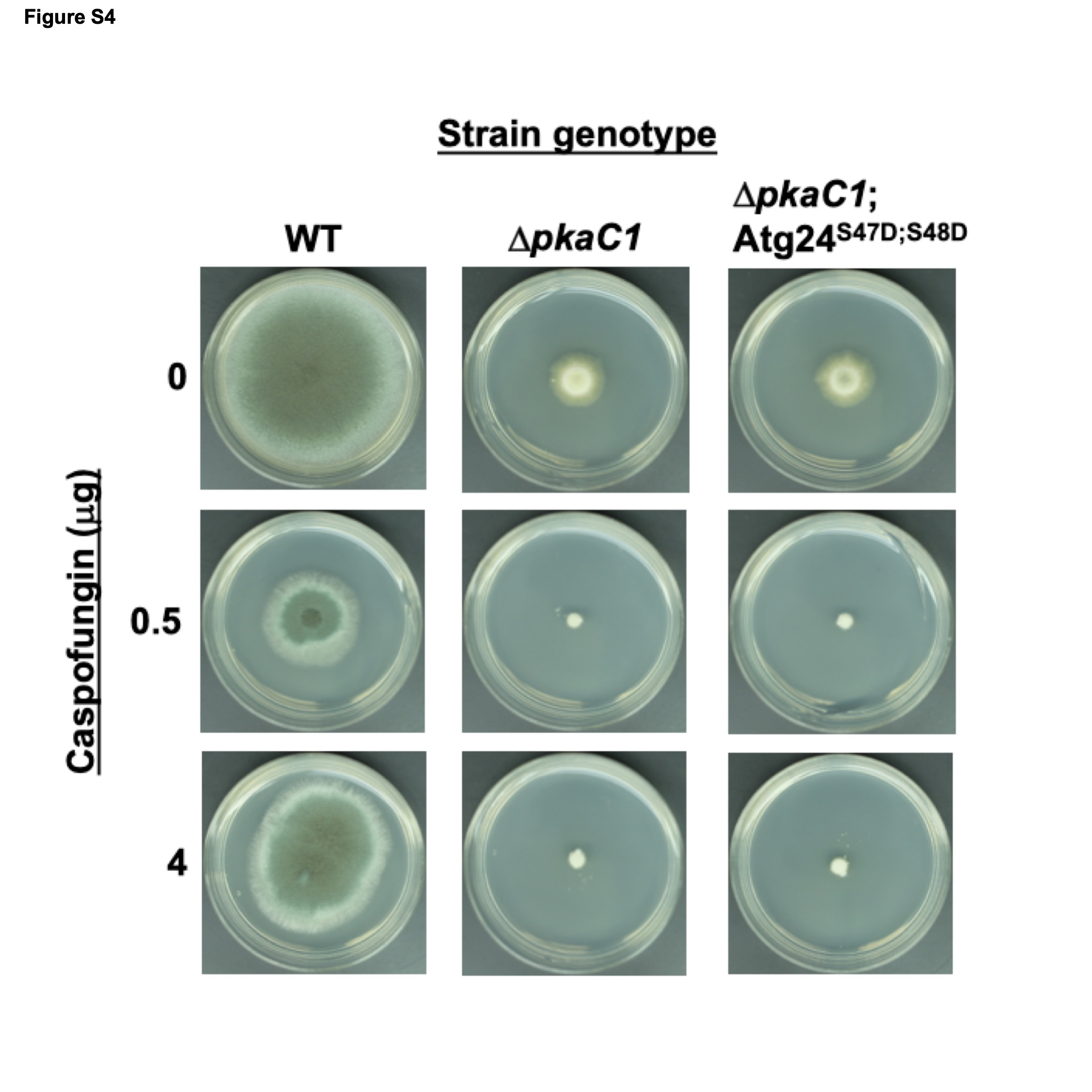

Supplement: FIG S4 [file mBio.02880-20-sf004.jpg]

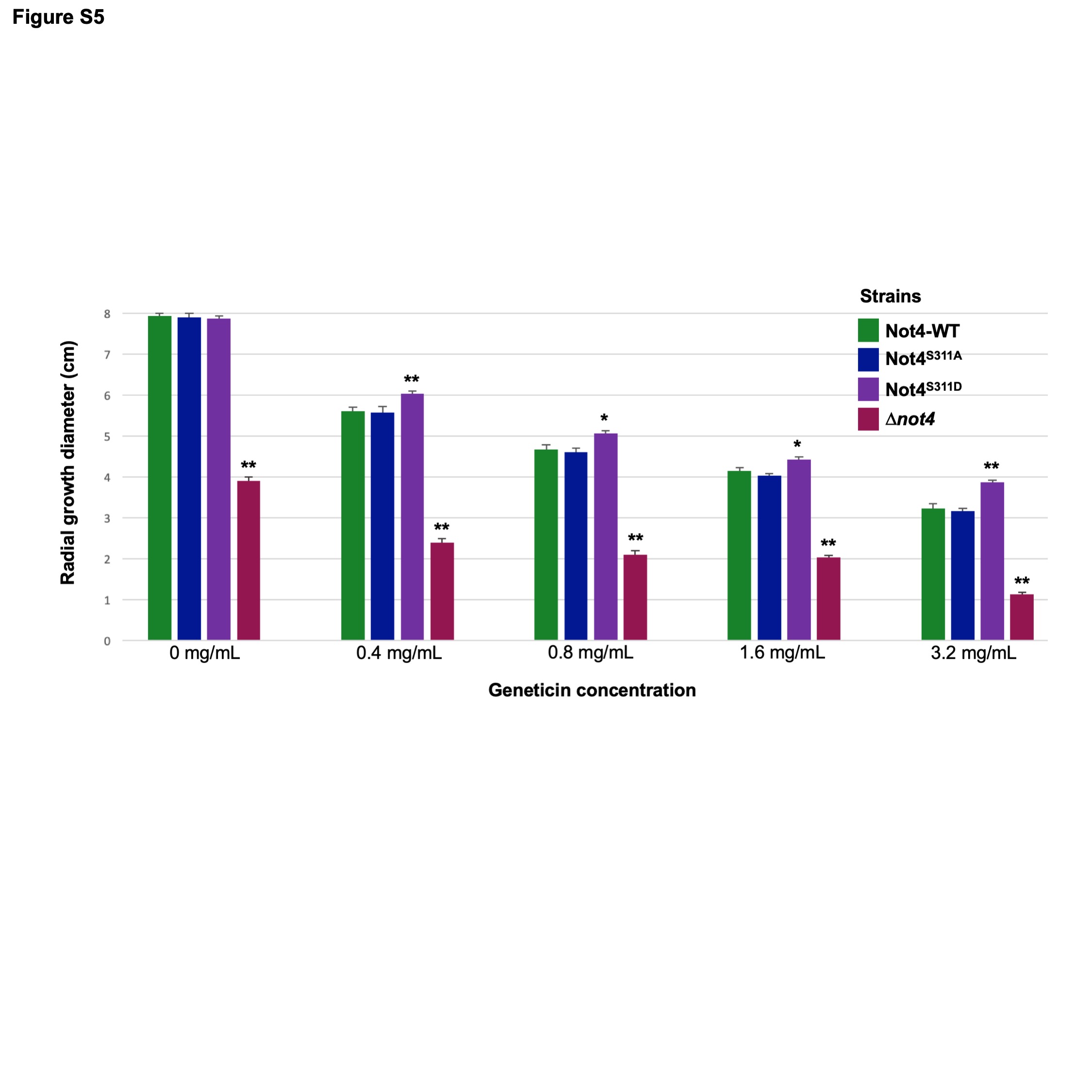

Supplement: FIG S5 [file mBio.02880-20-sf005.jpg]

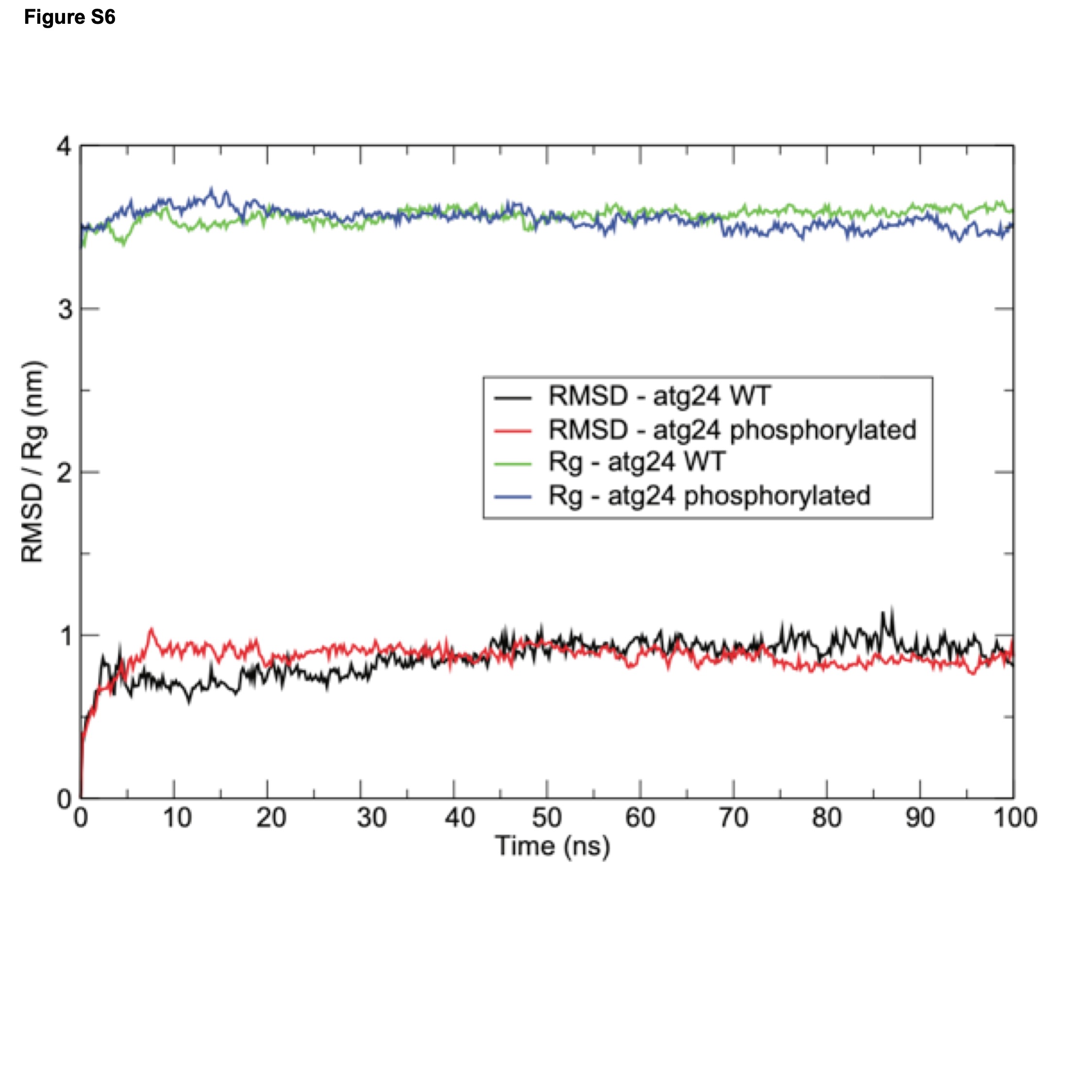

Supplement: FIG S6 [file mBio.02880-20-sf006.jpg]
