# Supplementary material for: The Protein Kinase A-Dependent Phosphoproteome of the Human Pathogen Aspergillus fumigatus Reveals Diverse Virulence-Associated Kinase Targets
Source: mBio. 2020 Dec 15;11(6):e02880-20. doi: 10.1128/mBio.02880-20 (PMC7773993; doi:10.1128/mBio.02880-20)
Supplement: TABLE S1 [file mBio.02880-20-st001.pdf]

Table S1. Proteins with decreased abundance in wild-type vs. ∆pkaC1 in open proteomic analysis

Category	No. of Proteins	Fold Enrichment	Enrichment P-value	Database
**Aerobic Respiration**				
Aerobic respiration	9	8.22	2.91E-06	GO Biological Process
Mitochondrial respiratory chain complex III	5	12.29	2.75E-04	GO Cellular Component
Glycolysis	7	7.95	1.33E-04	UniProtKB Keywords
Glycolysis / Gluconeogenesis	15	2.99	2.25E-04	KEGG Pathway
Glycolytic process	7	6.00	6.02E-04	GO Biological Process
Citrate cycle (TCA cycle)	9	3.03	0.00662005	KEGG Pathway
Tricarboxylic acid cycle	5	4.28	0.024784298	GO Biological Process
Tricarboxylic acid cycle	3	6.81	0.067673091	UniProtKB Keywords
Ubiquinone biosynthetic process	7	10.66	9.79E-06	GO Biological Process
Ubiquinone biosynthesis	4	12.11	0.002910421	UniProtKB Keywords
**Carbon Metabolism**				
Carbon metabolism	28	2.32	2.54E-05	KEGG Pathway
Glycine catabolic process	3	13.70	0.015049009	GO Biological Process
**Protein Synthesis**				
Ribosomal protein	20	3.03	2.67E-05	UniProtKB Keywords
Ribonucleoprotein	23	2.47	1.37E-04	UniProtKB Keywords
Structural constituent of ribosome	21	2.42	3.08E-04	GO Molecular Function
Translation	17	2.20	0.003693214	GO Biological Process
Aminoacyl-tRNA synthetase	6	2.95	0.050129488	UniProtKB Keywords
Elongation factor	3	3.89	0.17759984	UniProtKB Keywords
Translation elongation factor activity	3	2.64	0.314900496	GO Molecular Function
Protein biosynthesis	7	1.45	0.353976116	UniProtKB Keywords
Biosynthesis of amino acids	20	1.49	0.067393492	KEGG Pathway
**Fatty Acid Biosynthesis**				
Fatty acid hydroxylase	4	5.27	0.035868369	InterPro
Fatty acid biosynthetic process	4	2.61	0.192817087	GO Biological Process
**Secondary Metabolism**				
Biosynthesis of secondary metabolites	67	1.81	1.37E-07	KEGG Pathway
Biosynthesis of antibiotics	48	1.84	1.14E-05	KEGG Pathway
Secondary metabolites metabolism/transport	3	0.47	0.990914962	COG Ontology
**Ubiquitination**				
Ubiquitin-conjugating enzyme, active site	4	4.86	0.044499867	InterPro
Ubl conjugation pathway	6	2.95	0.050129488	UniProtKB Keywords
**Transcription/mRNA Processing**				
Zinc finger C2H2-type/integrase DNA-binding (	4	1.35	0.57724503	InterPro
RNA helicase, ATP-dependent, DEAD-box, co	3	2.37	0.362993373	InterPro
DEAD box	3	1.54	0.583798955	UniProtKB Seq. Feature
**Signaling**				
GTP binding	11	1.63	0.133305394	GO Molecular Function
Small GTP-binding protein domain	6	2.26	0.122893112	InterPro
Protein kinase-like domain	7	0.68	0.948400641	InterPro
**Redox Homeostasis**				
Cell redox homeostasis	5	2.14	0.200070944	GO Biological Process
**Protein Complex Assembly**				
WD repeat	4	2.08	0.301708814	UniProtKB Keywords
G-protein beta WD-40 repeat	4	1.37	0.562423314	InterPro
WD40	5	0.77	0.897386238	SMART
