# Supplementary material for: The Protein Kinase A-Dependent Phosphoproteome of the Human Pathogen Aspergillus fumigatus Reveals Diverse Virulence-Associated Kinase Targets
Source: mBio. 2020 Dec 15;11(6):e02880-20. doi: 10.1128/mBio.02880-20 (PMC7773993; doi:10.1128/mBio.02880-20)
Supplement: TABLE S2 [file mBio.02880-20-st002.pdf]

Table S2. Proteins with increased abundance in wild-type vs. ΔpkaC1 in open proteomic analysis

Category	No. of Proteins	Fold Enrichment	Enrichment P-value	Database
**Protein Synthesis**				
Protein biosynthesis	24	2.54	3.70E-05	UniProtKB Keywords
Amino acid biosynthesis	46	1.76	4.37E-05	KEGG Pathway
Amino acid biosynthetic process	6	2.91	0.042948638	GO Biological Process
Valine, leucine, isoleucine biosynthesis	9	2.50	0.015450645	KEGG Pathway
Aminoacyl-tRNA synthetase	18	4.53	5.81E-08	UniProtKB Keywords
Ribonucleoprotein	30	1.65	0.007122886	UniProtKB Keywords
Ribosomal protein	22	1.71	0.016265566	UniProtKB Keywords
Translation Initiation factors	12	2.03	0.029828306	UniProtKB Keywords
Aminotransferase	13	3.10	5.48E-04	UniProtKB Keywords
**Protein Degradation**				
Proteasome	17	4.06	9.58E-07	UniProtKB Keywords
Proteasome storage granule	8	3.65	0.003615316	GO Cellular Component
Ubiquitin-dependent protein catabolism	13	2.60	0.002232875	GO Biological Process
UBA-like Domain	7	2.94	0.024471874	InterPro
**Transcription**				
Transcription	21	0.96	0.701792189	UniProtKB Keywords
Transcription, RNA-templated	5	3.40	0.046551405	GO Biological Process
DNA-directed RNA polymerase II, core complex	5	4.10	0.0249327	GO Cellular Component
Transcription regulation	15	0.79	0.916317734	UniProtKB Keywords
Zinc finger, C2H2	7	0.82	0.870432834	InterPro
Zn(2)-C6 fungal-type DNA-binding domain	4	0.15	1	InterPro
RNA polymerase	8	1.48	0.281686349	KEGG Pathway
**Chromatin Structure**				
Ino80 complex	5	2.93	0.08030054	GO Cellular Component
Nucleosome core	4	6.20	0.019243174	UniProtKB Keywords
RSC complex	4	5.47	0.02699284	GO Cellular Component
Nucleosome	4	4.69	0.043032733	GO Cellular Component
Covalent chromatin modification	4	1.94	0.339498798	GO Biological Process
**mRNA Processing**				
mRNA processing	9	1.82	0.114466916	UniProtKB Keywords
mRNA splicing via spliceosome	10	2.19	0.029724004	GO Biological Process
Spliceosome	7	2.83	0.03078041	UniProtKB Keywords
U2-type prespliceosome	7	4.79	0.001527609	GO Cellular Component
**DNA Replication and Repair**				
DNA replication	7	2.50	0.053312378	UniProtKB Keywords
DNA repair	9	0.94	0.759538898	GO Biological Process
**Nitrogen Metabolism**				
Carbon-nitrogen hydrolase	4	3.19	0.12013871	InterPro
Nitrogen compound metabolic process	5	2.43	0.138129617	GO Biological Process
**Secondary Metabolism**				
Secondary metabolite biosynthesis	86	1.19	0.035173407	KEGG Pathway
Antibiotic biosynthesis	68	1.34	0.004591961	KEGG Pathway
Secondary metabolites biosynthesis/transport/cat	16	0.92	0.783346732	COG Ontology
**Cell Wall Organization**				
Cell wall	6	6.20	0.001229968	UniProtKB Keywords
Chitin synthases	3	2.99	0.264754664	InterPro
Chitin synthase activity	3	2.69	0.309110499	GO Molecular Function
Cell wall organization	6	1.13	0.626793054	GO Biological Process
**Signaling**				
Kinase	34	1.41	0.038863339	UniProtKB Keywords
Rho GTPase activation proteins	5	3.98	0.02762782	InterPro
Signal transduction	6	3.40	0.021831319	GO Biological Process
**Calcium Signaling**				
EF-hand-like domain	9	2.56	0.018405591	InterPro
Calcium ion binding	11	1.83	0.067220854	GO Molecular Function
Calcium/calmodulin-dependent protein kinase	4	2.66	0.182943817	InterPro
**Membrane Transport**				
ABC transporter, conserved site	7	1.27	0.479750883	InterPro
Mitochondrial carrier protein	3	1.49	0.613927685	InterPro
ATPase activity, coupled to transmembrane move	3	0.63	0.960905185	GO Molecular Function
Transmembrane transport	5	0.13	1	GO Biological Process
Major facilitator superfamily domain	7	0.17	1	InterPro
**Intracellular Transport**				
Motor activity	3	5.37	0.095954792	GO Molecular Function
Pleckstrin homology-like domain	9	1.79	0.119915669	InterPro
**Redox Homeostasis**				
Cell redox homeostasis	10	2.12	0.036163156	GO Biological Process
Thioredoxin-like fold	18	1.73	0.026580327	InterPro
Haem peroxidase	3	3.98	0.167228297	InterPro
Response to oxidative stress	4	2.72	0.171718781	GO Biological Process
