# Supplementary material for: The Protein Kinase A-Dependent Phosphoproteome of the Human Pathogen Aspergillus fumigatus Reveals Diverse Virulence-Associated Kinase Targets
Source: mBio. 2020 Dec 15;11(6):e02880-20. doi: 10.1128/mBio.02880-20 (PMC7773993; doi:10.1128/mBio.02880-20)
Supplement: TABLE S3 [file mBio.02880-20-st003.pdf]

**Table S3. Direct PKA target candidate proteins**

| Dataset | Interactome | Master Protein Accession | Master Protein Description | Expression Ratio | WT/ΔpkaC1 Phosphorylation Ratio | Adjusted Phos Ratio | Peptide Sequence | Modifications (Localization Probability %) | P-Value (WT vs. ΔpkaC1 |
|---|---|---|---|---|---|---|---|---|---|
| GluC TiOx | Yes | XP_751586.1 | conserved lysine-rich protein [Aspergillus fumigatus Af293] | 1.155196028 | 566.7615124 | 490.6193395 | AEAPKEAAAQSPAAETPAGKDKRRTSFFGNF | 1xPhospho [T25(95.4)] | 0.0458 |
| Trp Ab | No | XP_747974.1 | conserved hypothetical protein [Aspergillus fumigatus Af293] | 1.028606685 | 471.9180317 | 458.7934712 | RQSLASIR | 1xPhospho [S3(100)] | 0.0072 |
| Trp Ab | No | XP_750216.1 | PSP1 domain protein [Aspergillus fumigatus Af293] | 0.567836258 | 450.2880733 | 792.9892937 | KVSLTTGSLISGT | 1xPhospho [S3(99.4)] | 0.0006 |
| Trp Ab | Yes | XP_756056.1 | C2H2 transcription factor (AmdX) [Aspergillus fumigatus Af293] | NA | 398.9920656 | NA | KNSIASNSNSIRPR | 2xPhospho [S3(100); S6(73.1)] | 0.0000 |
| Trp Ab | Yes | XP_752292.1 | CUE domain protein [Aspergillus fumigatus Af293] | 0.934772908 | 336.4894576 | 359.9692018 | RGSDQSEEREYSFFDDDLPVIR | 2xPhospho [S3(100); S6(100)] | 0.0033 |
| Trp Ab | No | XP_752073.1 | snRNP assembly factor [Aspergillus fumigatus Af293] | 0.831022076 | 298.6491809 | 359.3757488 | RASGQEMEEHER | 1xPhospho [S3(100)] | 0.0004 |
| Trp Ab | No | XP_750917.1 | GPI-anchored cell wall protein Pst1 [Aspergillus fumigatus Af293] | NA | 150.4689104 | NA | SSVPASLLPGSGR | 1xPhospho [S2(84.6)] | 0.0019 |
| Trp Ab | No | XP_752463.1 | sphingosine kinase (SphK) [Aspergillus fumigatus Af293] | 0.920982266 | 147.9211785 | 160.6124069 | YAAGEPPRRPSEDTVAGSGGLPDLK | 1xPhospho [S11(99.9)] | 0.0004 |
| Trp Ab | No | XP_752411.2 | conserved hypothetical protein [Aspergillus fumigatus Af293] | NA | 131.3312913 | NA | RLSDIGEEDTGSSPR | 1xPhospho [S3(100)] | 0.0018 |
| Trp Ab | No | XP_748072.1 | MFS multidrug transporter [Aspergillus fumigatus Af293] | NA | 113.011456 | NA | RFSLGTGQGQVDPLDLPLR | 1xPhospho [S3(99.9)] | 0.0013 |
| Trp Ab | No | XP_750771.1 | CCR4-NOT core complex subunit Not4 [Aspergillus fumigatus Af293] | 0.937117338 | 101.6205273 | 108.4394911 | RASLAGSQASQSPRPVHAT | 1xPhospho [S3(100)] | 0.0059 |
| Trp Ab | No | XP_755804.1 | CCAAT-binding transcription factor subunit HAPB [Aspergillus fumigatus Af293] | 0.528527808 | 82.18512311 | 155.4982007 | RKSSEVNDDNANSVK | 1xPhospho [S4(99.2)] | 0.0081 |
| Trp Ab | No | XP_755144.1 | conserved hypothetical protein [Aspergillus fumigatus Af293] | 1.671742266 | 80.03529784 | 47.87538095 | RHSSSDPYFTPR | 2xPhospho [S3(100); S4(96.8)] | 0.0036 |
| Trp Ab | No | XP_746343.1 | MFS multidrug transporter [Aspergillus fumigatus Af293] | NA | 54.56480073 | NA | SHTRRNTNDLGLVPTETK | 1xPhospho [T7(97.9)] | 0.0019 |
| Trp Ab | No | XP_753399.1 | arrestin (or S-antigen), N-terminal domain protein [Aspergillus fumigatus Af293] | NA | 52.99723455 | NA | RLSFNMDHLIHPHR | 1xPhospho [S3(100)] | 0.0008 |
| Trp Ab | No | XP_001481719 | conserved hypothetical protein [Aspergillus fumigatus Af293] | NA | 49.82231463 | NA | SMKPDIDDEGFVTDGGPVR | 1xPhospho [S1(100)] | 0.0076 |
| Trp Ab | No | XP_747824.1 | actin associated protein Wsp1 [Aspergillus fumigatus Af293] | 1.112668858 | 44.86743503 | 40.32415818 | KVSAPPAPPSR | 1xPhospho [S3(100)] | 0.0152 |
| Trp Ab | No | XP_752174.2 | conserved hypothetical protein [Aspergillus fumigatus Af293] | NA | 43.8102387 | NA | RNSLDTNASVR | 1xPhospho [S3(100)] | 0.0004 |
| Trp Ab | No | XP_747815.1 | 2-dehydropantoate 2-reductase [Aspergillus fumigatus Af293] | 1.203974345 | 42.53418313 | 35.32814741 | SRRNSQSPATPYGGPGPR | 1xPhospho [S5(99.8)] | 0.0027 |
| Trp Ab | No | XP_752395.1 | chromodomain helicase (Chd1) [Aspergillus fumigatus Af293] | 0.850549652 | 40.20466913 | 47.26904425 | KSSDNGNSDEMLR | 1xPhospho [S3(99.8)] | 0.0011 |
| Trp Ab | No | XP_754486.2 | bZIP transcription factor (AtfA) [Aspergillus fumigatus Af293] | 0.695277256 | 37.98630389 | 54.63475698 | DADRRGSQNVNGPVASSR | 1xPhospho [S7(100)] | 0.0027 |
| Trp Ab | No | XP_754055.1 | 1,3-beta-glucan biosynthesis protein [Aspergillus fumigatus Af293] | 1.186174326 | 34.74736891 | 29.2936444 | VQRRESSQGTTAVS | 1xPhospho [S6(87.6)] | 0.0134 |
| Trp Ab | No | XP_753035.1 | conserved hypothetical protein [Aspergillus fumigatus Af293] | 1.031884426 | 34.6360088 | 33.56578308 | RRSWGNIDNAGLINL | 1xPhospho [S3(100)] | 0.0014 |
| Trp Ab | No | XP_751518.1 | conserved hypothetical protein [Aspergillus fumigatus Af293] | NA | 33.43927907 | NA | RMSVQGFDQEGNPCTK | 1xCarbamidomethyl [C14]; 1xPhospho [S3(100)] | 0.0413 |
| Trp Ab | No | XP_752259.1 | IQ calmodulin-binding motif domain protein [Aspergillus fumigatus Af293] | NA | 28.98576852 | NA | RNSDIEIQLPDAGPTVAPESVTEPDVDMLPDEQKPPLNLPAR | 1xPhospho [S3(100)] | 0.0281 |
| Trp Ab | No | XP_754510.1 | Golgi complex component Cog3 [Aspergillus fumigatus Af293] | NA | 28.85827754 | NA | RQTAPSGPPK | 1xPhospho [T3(100)] | 0.0101 |
| Trp Ab | No | XP_755021.1 | conserved hypothetical protein [Aspergillus fumigatus Af293] | 0.78843947 | 27.79682197 | 35.25549267 | RHSKPDASEGQWTHDLHETVAGDEPTNKPSAVPE | 1xPhospho [S3(99.7)] | 0.0015 |
| Trp Ab | Yes | XP_752473.1 | serine/threonine protein kinase Kin1 [Aspergillus fumigatus Af293] | 0.793951621 | 27.67430492 | 34.85641214 | RRISFGGLLNHDDSK | 1xPhospho [S4(100)] | 0.0033 |
| Trp Ab | No | XP_746920.1 | conserved hypothetical protein [Aspergillus fumigatus Af293] | NA | 26.2217905 | NA | RRESSADLPWELEHMR | 1xPhospho [S4(99.3)] | 0.0247 |
| Trp Ab | Yes | XP_751536.1 | vacuolar targeting protein Atg24 [Aspergillus fumigatus Af293] | 0.798989249 | 25.2522389 | 31.60522988 | RRMSSVHEDPPQAGPLADAVDLAGIGDGVLEC | 1xCarbamidomethyl [C32]; 1xPhospho [S4(82.6)] | 0.0020 |
| Trp Ab | No | XP_749671.1 | conserved hypothetical protein [Aspergillus fumigatus Af293] | NA | 25.17118236 | NA | SADDVDTSAPELAPHRRGSDAR | 2xPhospho [S1(100); S19(100)] | 0.0341 |
| Trp TiOx | No | XP_748000.1 | PHD transcription factor (Rum1) [Aspergillus fumigatus Af293] | 1.499764276 | 25.09028898 | 16.72948835 | SLESQTPRPA | 1xPhospho [S1(100)] | 0.0010 |
| Trp Ab | No | XP_755122.1 | Patatin-like serine hydrolase [Aspergillus fumigatus Af293] | NA | 23.71798477 | NA | QRKPSYHVR | 1xPhospho [S5(100)] | 0.0432 |
| Trp Ab | No | XP_755953.1 | DNA-directed RNA polymerase III RPC4 [Aspergillus fumigatus Af293] | 0.552230618 | 23.46502929 | 42.49135874 | RLTPKTPEPEPAPAPAPPSKPAVAK | 2xPhospho [T3(100); T6(100)] | 0.0214 |
| Trp Ab | Yes | XP_754847.1 | plasma membrane H+-ATPase Pma1 [Aspergillus fumigatus Af293] | 1.265941217 | 22.93246244 | 18.11495047 | RRISYAPDVENGDHSR | 1xPhospho [S4(100)] | 0.0287 |
| Trp Ab | No | XP_752860.2 | endosomal SPRY domain protein [Aspergillus fumigatus Af293] | NA | 20.04081675 | NA | RGSDITGDLPNQSSPPIPSYDAAVGNQADNTLRPDGDH | 1xPhospho [S3(98)] | 0.0199 |
| GluC Ab | No | XP_747211.2 | cleavage and polyadenylation specificity factor subunit A, putative [Aspergillus fumigatus Af293] | 0.822540278 | 18.30168735 | 22.25020201 | DAYEDDLYTAEPETPALGRRPSAETT | 1xPhospho [S22(98.6)] | 0.0003 |
| Trp Ab | No | XP_755203.1 | nuclear distribution protein NudE [Aspergillus fumigatus Af293] | NA | 18.16840193 | NA | KRTPSGISGIPAPR | 1xPhospho [T3(99.7)] | 0.0025 |
| Trp Ab | No | XP_749131.1 | hypothetical protein AFUA_7G03770 [Aspergillus fumigatus Af293] | 0.178138485 | 17.6721067 | 99.20431687 | RPSLSSGFFTDYLSR | 1xPhospho [S3(99.7)] | 0.0042 |
| Trp Ab | No | XP_748003.1 | SH3 domain protein (Cyk3) [Aspergillus fumigatus Af293] | NA | 17.15680865 | NA | RGSEDILPPEDAEEPPPPPK | 1xPhospho [S3(100)] | 0.0372 |
| Trp Ab | No | XP_751112.1 | GTPase activating protein (BUD2/CLA2) [Aspergillus fumigatus Af293] | 1.438713314 | 16.82913958 | 11.69735445 | KPSRSHSNPVPAPAVSDYYTE | 1xPhospho [S3(78.6)] | 0.0000 |
| Trp Ab | No | XP_753053.1 | sphinganine hydroxylase Sur2 [Aspergillus fumigatus Af293] | 0.97294912 | 16.00166124 | 16.44655503 | RKTVTLSPHVDSLK | 1xPhospho [T3(100)] | 0.0174 |
| Trp Ab | No | XP_750272.2 | high affinity cAMP phosphodiesterase [Aspergillus fumigatus Af293] | NA | 15.87387457 | NA | GSPIPFVPSRKSSSAVPDLATPSVSGTSPDADGSEHAGDFER | 3xPhospho [S9(96.6); S13(69.9); S34(100)] | 0.0428 |
| Trp Ab | No | XP_754770.1 | conserved hypothetical protein [Aspergillus fumigatus Af293] | 0.866602219 | 15.69229053 | 18.10783561 | STDENYHVGR | 1xPhospho [T2(91.1)] | 0.0407 |
| Trp Ab | No | XP_751660.1 | Leucine Rich Repeat domain protein [Aspergillus fumigatus Af293] | NA | 13.44092871 | NA | RRPSFGSIKEDVDGIAQSF | 1xPhospho [S4(99.9)] | 0.0153 |
| Trp Ab | No | XP_755853.1 | conserved hypothetical protein [Aspergillus fumigatus Af293] | 0.933824131 | 13.36875018 | 14.3161327 | EGEETVRRHSVVPAEIVGGCGGRPPLR | 1xCarbamidomethyl [C20]; 1xPhospho [S10(99.9)] | 0.0028 |
| Trp Ab | No | XP_001481667 | RING finger protein [Aspergillus fumigatus Af293] | NA | 12.77743977 | NA | RLSSASALPSNPLPQVDEHPLMQPSVEPAPESTADRIPFQPSHQR | 1xPhospho [S3(65.5)] | 0.0337 |
| Trp Ab | No | XP_755379.1 | cytosolic regulator Pianissimo [Aspergillus fumigatus Af293] | NA | 11.83237511 | NA | RRGSSNVELDELSSTEER | 1xPhospho [S4(98)] | 0.0401 |
| Trp Ab | No | XP_752218.1 | conserved hypothetical protein [Aspergillus fumigatus Af293] | NA | 11.10510772 | NA | RHSIAASDPASSAIGSEDTR | 1xPhospho [S3(100)] | 0.0184 |
| Trp Ab | No | XP_749463.1 | bZIP transcription factor [Aspergillus fumigatus Af293] | NA | 10.77727614 | NA | MASRKPSASILVPR | 1xPhospho [S7(99.7)] | 0.0305 |
| Trp Ab | No | XP_001481443. | C6 transcription factor [Aspergillus fumigatus Af293] | 0.495505398 | 10.45895072 | 21.10764233 | RGSGSGFEYPDPTNLAPVTPATSAPAYQAHPAPSPYYPPPPQHDR | 1xPhospho [S3(71.5)] | 0.0105 |
| Trp TiOx | No | XP_750055.1 | DEAD box helicase Mph1 [Aspergillus fumigatus Af293] | NA | 8.876608182 | NA | KPTPEPVEVPALEEVLLTPR | 1xPhospho [T3(100)] | 0.0146 |
| GluC Ab | Yes | XP_752178.1 | dDENN domain protein [Aspergillus fumigatus Af293] | NA | 8.60581712 | NA | RRLSNYNSVIPAPQPLE | 1xPhospho [S4(100)] | 0.0324 |
| GluC Ab | No | XP_747268.1 | conserved hypothetical protein [Aspergillus fumigatus Af293] | NA | 8.306328624 | NA | RRPTGVGLWDE | 1xPhospho [T4(100)] | 0.0079 |
| TrpC TiO2 | No | XP_749288.1 | protein kinase [Aspergillus fumigatus Af293] | NA | 7.96585114 | NA | KSSLPLDIGLPK | 1xPhospho [S3(100)] | |
| GluC Ab | No | XP_753062.1 | DEAD/DEAH box helicase [Aspergillus fumigatus Af293] | 0.818718534 | 7.951063668 | 9.711596033 | KTRKDSAVAPIGSK | 1xPhospho [S6(100)] | 0.0047 |
| Trp Ab | No | XP_752895.2 | conserved hypothetical protein [Aspergillus fumigatus Af293] | 0.968634683 | 7.752210941 | 8.003234939 | RRDSIQCIHEPFGDAFYYGPER | 1xCarbamidomethyl [C7]; 1xPhospho [S4(100)] | 0.0278 |
| Trp Ab | No | XP_746762.2 | conserved hypothetical protein [Aspergillus fumigatus Af293] | NA | 7.623195708 | NA | RHSYRDPTSSSSSGAHAA | 1xPhospho [S3(100)] | 0.0101 |
| Trp Ab | No | XP_751511.1 | conserved hypothetical protein [Aspergillus fumigatus Af293] | NA | 7.385480831 | NA | KSSIVTVDECPPCLR | 2xCarbamidomethyl [C10; C13]; 1xPhospho [S3(95.3)] | 0.0040 |
| Trp Ab | No | XP_752831.1 | Fibronectin type III domain protein [Aspergillus fumigatus Af293] | 0.914888913 | 7.001339218 | 7.652665932 | RLSGLFGFHR | 1xPhospho [S3(100)] | 0.0010 |
| Trp Ab | No | XP_751003.1 | protein kinase (VPS15) [Aspergillus fumigatus Af293] | NA | 6.687598438 | NA | RGSVGRQSPGQDSPSTPTEAGSPAIK | 5xPhospho [S3(100); S8(100); S13(100); S15(79.1); T/S] | 0.0126 |
| Trp Ab | No | XP_751987.1 | hypothetical protein AFUA_4G08380 [Aspergillus fumigatus Af293] | 0.819991471 | 6.645029997 | 8.103779409 | RRSQSNVTEPTASGSGTTSSK | 1xPhospho [S3(86)] | 0.0043 |
| Trp Ab | No | XP_749681.1 | conserved hypothetical protein [Aspergillus fumigatus Af293] | 0.900717513 | 6.52614124 | 7.245491676 | RRGSQVNGIMDPSLQNK | 1xPhospho [S4(100)] | 0.0018 |
| Trp Ab | No | XP_754248.1 | conserved hypothetical protein [Aspergillus fumigatus Af293] | NA | 6.443826255 | NA | RLSSQSSEAADR | 1xPhospho [S3(98.3)] | 0.0166 |
| Trp Ab | No | XP_749920.1 | conserved serine-rich protein [Aspergillus fumigatus Af293] | 0.389958698 | 6.392461889 | 16.39266396 | SLEEEQPNKEPR | 1xPhospho [S1(100)] | 0.0005 |
| Trp Ab | No | XP_747212.2 | transcriptional regulator PacG/VIB-1 [Aspergillus fumigatus Af293] | NA | 6.366994668 | NA | KLSHSVSQAPSR | 1xPhospho [S3(99.9)] | 0.0300 |
| Trp Ab | No | XP_755141.1 | involucrin repeat protein [Aspergillus fumigatus Af293] | 0.589950758 | 6.251134843 | 10.59602815 | RKNTAETPVQNEAVHTADPISSIEGVPEPGPEATTT | 1xPhospho [T4(97.4)] | 0.0307 |
| Trp Ab | No | XP_754733.1 | conserved hypothetical protein [Aspergillus fumigatus Af293] | NA | 6.001498278 | NA | SRRVTNDDIDEIFSDESSFYGDDAEK | 1xPhospho [T5(100)] | 0.0098 |
| Trp Ab | No | XP_748845.2 | conserved hypothetical protein [Aspergillus fumigatus Af293] | NA | 5.925899481 | NA | RHSFYYLPDEADSAGGQPMTPER | 1xPhospho [S3(100)] | 0.0003 |
| Trp Ab | No | XP_755622.1 | PtaB protein [Aspergillus fumigatus Af293] | NA | 5.765250362 | NA | RASTVKVENDDTGGPEVNGT | 2xPhospho [S3(100); T4(100)] | 0.0494 |
| Trp Ab | Yes | XP_753922.1 | RNP domain protein [Aspergillus fumigatus Af293] | 0.75578594 | 5.700597685 | 7.542608803 | KVSVQLA | 1xPhospho [S3(100)] | 0.0366 |

| | | | | | | | | | |
|---|---|---|---|---|---|---|---|---|---|
| GluC Ab | No | XP_753388.1 | UPF0052 domain protein [Aspergillus fumigatus Af293] | NA | 5.688329387 | NA | ALQPRPGSRRPSLLLADGASDLEDTEPSDASDAASYE | 2xPhospho [S8(100); S12(100)] | 0.0003 |
| Trp Ab | No | XP_752603.1 | carnitine acetyl transferase [Aspergillus fumigatus Af293] | 0.249612442 | 5.362708922 | 21.48414108 | RGTDTPTTDSGDIEDD | 1xPhospho [T3(100)] | 0.0289 |
| Trp Ab | No | XP_755624.1 | conserved hypothetical protein [Aspergillus fumigatus Af293] | NA | 5.178466253 | NA | HRRGSSHGSHPEAHDGQPK | 2xPhospho [S5(95.7); S6(82)] | 0.0154 |
| Trp Ab | Yes | XP_755648.1 | conserved hypothetical protein [Aspergillus fumigatus Af293] | 1.699647529 | 5.045318387 | 2.968449811 | RGSPYSQASDQGQK | 1xPhospho [S3(100)] | 0.0399 |
| Trp TiOx | No | XP_754440.1 | conserved hypothetical protein [Aspergillus fumigatus Af293] | 1.638201968 | 4.901844439 | 2.992210078 | SLQTDFSSIGPLKPGGTVGFL | 1xPhospho [S1(100)] | 0.0361 |
| GluC Ab | Yes | XP_752949.1 | conserved hypothetical protein [Aspergillus fumigatus Af293] | 1.161999051 | 4.368737525 | 3.759673917 | SGLGPTLRKSSTISLT | 1xPhospho [S11(82)] | 0.0036 |
| Trp Ab | Yes | XP_753687.1 | conserved hypothetical protein [Aspergillus fumigatus Af293] | 0.741597522 | 4.345017923 | 5.858997359 | NSRRPSISSAIDESVPLQLR | 1xPhospho [S6(99.5)] | 0.0150 |
| Trp Ab | No | XP_747234.1 | AAA family ATPase [Aspergillus fumigatus Af293] | 0.97191962 | 4.320147657 | 4.444963936 | RHSNDDITSDDDDILDDVDELDFDDEDEEDPNAPK | 2xPhospho [S3(100); T8(90.6)] | 0.0486 |
| Trp Ab | Yes | XP_755446.2 | NOT2 family protein [Aspergillus fumigatus Af293] | 0.453620035 | 4.316358573 | 9.51536141 | NVAADLGQDRRESLMQSA | 1xPhospho [S13(100)] | 0.0048 |
| Trp Ab | No | XP_755142.1 | CorA family metal ion transporter [Aspergillus fumigatus Af293] | NA | 4.303845491 | NA | RQSFLAPEESHNGAAASGADADAM | 1xPhospho [S3(100)] | 0.0042 |
| Trp Ab | No | XP_755108.1 | nuclear migration protein [Aspergillus fumigatus Af293] | 0.87215197 | 4.299609188 | 4.929885315 | SFGGVGEPK | 1xPhospho [S1(100)] | 0.0115 |
| Trp Ab | No | XP_750270.2 | G-protein complex gamma subunit GpgA [Aspergillus fumigatus Af293] | 0.464411237 | 4.221040069 | 9.089013639 | KQSVADLK | 1xPhospho [S3(100)] | 0.0276 |
| Trp Ab | No | XP_749349.1 | CUE domain protein [Aspergillus fumigatus Af293] | NA | 4.095775193 | NA | RDSDLTADTILADR | 1xPhospho [S3(100)] | 0.0349 |
| Trp Ab | No | XP_755976.2 | translation initiation factor 4B [Aspergillus fumigatus Af293] | 0.866432484 | 4.004796231 | 4.622167686 | RSSPAWGEGR | 1xPhospho [S3(99.9)] | 0.0222 |
| Trp Ab | No | XP_747882.1 | conserved hypothetical protein [Aspergillus fumigatus Af293] | 1.228153111 | 3.847293672 | 3.132584721 | ARRFSFFDSITR | 1xPhospho [S5(100)] | 0.0083 |
| Trp TiOx | Yes | XP_752510.1 | myosin class II heavy chain (MHC) [Aspergillus fumigatus Af293] | 0.846299298 | 3.80102581 | 4.491349359 | RASYDHDAEPADYADA | 1xPhospho [S3(100)] | 0.0381 |
| GluC Ab | Yes | XP_755687.2 | CCCH zinc finger and RRM domain protein [Aspergillus fumigatus Af293] | 0.733874309 | 3.778570239 | 5.148797542 | GAPMGGAAPAHTGPDGLNFNRKRTYHE | 1xPhospho [T24(100)] | 0.0363 |
| Trp Ab | No | XP_001481632 | Mis12-Mtw1 family protein [Aspergillus fumigatus Af293] | NA | 3.768760488 | NA | RASSLIDSGASNALPHK | 1xPhospho [S3(90)] | 0.0000 |
| Trp Ab | No | XP_755365.1 | SH3 domain protein [Aspergillus fumigatus Af293] | 0.866159725 | 3.728350059 | 4.304460197 | RMSRPPPPIPTNPPMSPQFQGR | 1xOxidation [M2]; 1xPhospho [S3(100)] | 0.0124 |
| Trp Ab | No | XP_747653.2 | pheromone-dependent cell cycle arrest protein Far11 [Aspergillus fumigatus Af293] | 0.769816209 | 3.498716988 | 4.544873108 | HRRDTDPGTSSVRPPSPQEALPELVEGAHRPEVDEL | 2xPhospho [T5(99.7); S11(60.6)] | 0.0168 |
| Trp Ab | No | XP_750333.1 | TBP associated factor (Mot1) [Aspergillus fumigatus Af293] | 1.42282709 | 3.405495683 | 2.393471214 | RASENVTTPSVATPYPIK | 1xPhospho [S3(100)] | 0.0143 |
| Trp Ab | No | XP_750682.1 | PH domain protein [Aspergillus fumigatus Af293] | 0.953939472 | 3.386864211 | 3.550397387 | RASLSDDEAVPGSDTNETTGLLLERL | 2xPhospho [S3(100); S5(100)] | 0.0334 |
| Trp Ab | No | XP_753868.1 | SH3 domain signalling protein [Aspergillus fumigatus Af293] | 0.787873639 | 3.310631199 | 4.201982445 | RRSNGQGAGAGASHPVPPLQPAAPSICR | 1xCarbamidomethyl [C27]; 1xPhospho [S3(100)] | 0.0121 |
| Trp TiOx | No | XP_754558.1 | conserved hypothetical protein [Aspergillus fumigatus Af293] | 0.857752912 | 3.304540401 | 3.852555153 | SLQLPADEQGIPEIGQR | 1xPhospho [S1(100)] | 0.0143 |
| Trp Ab | No | XP_746653.1 | calcium permease family membrane transporter [Aspergillus fumigatus Af293] | NA | 3.139104728 | NA | RRNSIDSVSEQDSLLGR | 4xPhospho [S4(100); S7(100); S9(100); S13(100)] | 0.0069 |
| Trp Ab | No | XP_750137.1 | Cut9 interacting protein Scn1 [Aspergillus fumigatus Af293] | NA | 3.035469959 | NA | RLSVEGAHAESDAEDEQVQK | 2xPhospho [S3(100); S11(100)] | 0.0132 |
| Trp Ab | No | XP_751970.1 | regulatory protein Ral2 [Aspergillus fumigatus Af293] | NA | 3.020732522 | NA | VRRDTDAQLRPH | 1xPhospho [T5(100)] | 0.0224 |
| Trp Ab | No | XP_750273.1 | PHD finger and BAH domain protein (Snt2) [Aspergillus fumigatus Af293] | NA | 2.995447051 | NA | RSSAINGVVT | 1xPhospho [S3(93.6)] | 0.0066 |
| Trp Ab | No | XP_747928.1 | conserved hypothetical protein [Aspergillus fumigatus Af293] | 1.12912297 | 2.94207493 | 2.605628448 | LGRRMTSQDAVMR | 1xPhospho [T6(99.6)] | 0.0255 |
| Trp Ab | No | XP_755655.1 | universal stress protein family domain protein [Aspergillus fumigatus Af293] | NA | 2.91720239 | NA | RSSDNSDQISIGK | 1xPhospho [S3(100)] | 0.0046 |
| Trp Ab | No | XP_749452.1 | cyclic nucleotide-binding domain protein [Aspergillus fumigatus Af293] | NA | 2.807955248 | NA | ETSTPSADLTSPVRRGSK | 1xPhospho [S17(87)] | 0.0402 |
| Trp Ab | No | XP_756003.2 | tRNA nucleotidyltransferase [Aspergillus fumigatus Af293] | NA | 2.78393588 | NA | RISVTAPHEGASDTMAT | 1xPhospho [S3(100)] | 0.0375 |
| Trp Ab | No | XP_755578.1 | conserved hypothetical protein [Aspergillus fumigatus Af293] | 1.147717939 | 2.754861567 | 2.400294945 | QRRSTGAFSTPR | 1xPhospho [T5(87.4)] | 0.0165 |
| Trp Ab | No | XP_752334.1 | HLH transcription factor (GlcD gamma) [Aspergillus fumigatus Af293] | 0.626634907 | 2.687650778 | 4.289021802 | RATAGNITMGLPIEEK | 1xPhospho [T3(100)] | 0.0024 |
| Trp Ab | No | XP_753813.1 | C3HC4 finger protein [Aspergillus fumigatus Af293] | NA | 2.657693731 | NA | RLSTSQVPR | 1xPhospho [S3(99.3)] | 0.0001 |
| Trp TiOx | No | XP_755834.1 | serine threonine protein kinase [Aspergillus fumigatus Af293] | 1.06029647 | 2.534296978 | 2.3901777 | RLSEPALADDFGSF | 1xPhospho [S3(100)] | 0.0009 |
| Trp Ab | No | XP_752048.2 | cation chloride cotransporter [Aspergillus fumigatus Af293] | NA | 2.532586642 | NA | RESGRQDSGPDNVAAN | 1xPhospho [S3(99.9)] | 0.0074 |
| Trp Ab | No | XP_755759.1 | conserved hypothetical protein [Aspergillus fumigatus Af293] | NA | 2.502273644 | NA | RLSYKYEDEEPDSAHAA | 1xPhospho [S3(100)] | 0.0039 |
| Trp Ab | No | XP_751989.1 | RNA polymerase II mediator complex component Med8 [Aspergillus fumigatus Af293] | NA | 2.480416028 | NA | RRSGAGAGLEFDIAAPAPGSR | 1xPhospho [S3(100)] | 0.0028 |
| Trp Ab | No | XP_748914.1 | C2H2 finger domain protein [Aspergillus fumigatus Af293] | NA | 2.388427238 | NA | RHSLEDDGVSESR | 1xPhospho [S3(100)] | 0.0366 |
| Trp Ab | No | XP_752665.1 | Avl9 protein [Aspergillus fumigatus Af293] | 0.860292696 | 2.377515708 | 2.763612569 | RESGDFEPPNAK | 1xPhospho [S3(100)] | 0.0070 |
| Trp Ab | No | XP_753339.1 | conserved hypothetical protein [Aspergillus fumigatus Af293] | NA | 2.34718059 | NA | RFSGLAFAN | 1xPhospho [S3(100)] | 0.0154 |
| Trp Ab | No | XP_753865.1 | nuclear migration protein (ApsA) [Aspergillus fumigatus Af293] | 0.956679077 | 2.303812469 | 2.408135104 | MRPTADSRRPSEAILDVPAKPA | 2xPhospho [S7(97.3); S11(100)] | 0.0310 |
| Trp Ab | No | XP_752739.1 | WD repeat protein [Aspergillus fumigatus Af293] | 0.776739583 | 2.252728227 | 2.900236163 | RSSLVVADLPIK | 1xPhospho [S3(99.4)] | 0.0110 |
| GluC Ab | No | XP_752476.1 | calcium channel subunit Cch1 [Aspergillus fumigatus Af293] | NA | 2.181754273 | NA | SARRRESSSDLINVPLDDFGPQEAE | 1xPhospho [S7(70.1)] | 0.0276 |
| Trp Ab | No | XP_753735.1 | ER membrane protein Wsc4 [Aspergillus fumigatus Af293] | 0.966418768 | 2.171730887 | 2.247194445 | GNGRRDSDVSLQDNEDYSRPVLR | 1xPhospho [S7(100)] | 0.0441 |
| Trp Ab | No | XP_753323.1 | C6 transcription factor [Aspergillus fumigatus Af293] | NA | 2.166654347 | NA | RKPSEIEDNVDGVLLR | 1xPhospho [S4(100)] | 0.0340 |
| Trp Ab | No | XP_751790.1 | RING and UBP finger domain protein [Aspergillus fumigatus Af293] | 0.876137173 | 2.147618015 | 2.451234898 | RGSQGAAYGDEPAECSVC | 2xCarbamidomethyl [C15; C18]; 1xPhospho [S3(100)] | 0.0264 |
| Trp Ab | No | XP_748169.1 | MYB DNA-binding domain protein [Aspergillus fumigatus Af293] | NA | 2.083158878 | NA | RDSIRPDLAASRPDPVK | 1xPhospho [S3(100)] | 0.0082 |
| GluC Ab | Yes | XP_754424.1 | C2H2 transcription factor PacC [Aspergillus fumigatus Af293] | 0.778598338 | 2.059713419 | 2.64541204 | ERRPSIQMDTSHDGKEDGE | 1xPhospho [S5(100)] | 0.0128 |
| Trp TiOx | No | XP_747591.2 | MBOAT family protein [Aspergillus fumigatus Af293] | 0.963750534 | 2.055949536 | 2.133279791 | RGSVVGVPTGEELK | 1xPhospho [S3(100)] | 0.0068 |
| Trp Ab | No | XP_755083.1 | conserved hypothetical protein [Aspergillus fumigatus Af293] | NA | 2.036178596 | NA | RMSGQNPTELK | 1xPhospho [S3(100)] | 0.0365 |
| Trp Ab | No | XP_755139.1 | C6 finger domain protein [Aspergillus fumigatus Af293] | NA | 2.017023631 | NA | RASNPPQPSLPPHGYPVIPNR | 1xPhospho [S3(100)] | 0.0169 |
| Trp TiOx | Yes | XP_752507.1 | cell wall biogenesis protein phosphatase Ssd1 [Aspergillus fumigatus Af293] | 0.824215075 | 1.962694928 | 2.38128977 | RHSLALPEAK | 1xPhospho [S3(100)] | 0.0341 |
| Trp TiOx | No | XP_752865.1 | gelsolin repeat protein [Aspergillus fumigatus Af293] | 0.774801503 | 1.763142952 | 2.275606005 | NTAGEIDAPAK | 1xPhospho [T2(100)] | 0.0262 |
| GluC Ab | No | XP_748012.1 | conserved hypothetical protein [Aspergillus fumigatus Af293] | 0.866865379 | 1.830605733 | 2.111753196 | AEHRRQSTLNEPTTAPSENPSLK | 1xPhospho [S7(97.6)] | 0.0132 |
