# Supplementary material for: The Protein Kinase A-Dependent Phosphoproteome of the Human Pathogen Aspergillus fumigatus Reveals Diverse Virulence-Associated Kinase Targets
Source: mBio. 2020 Dec 15;11(6):e02880-20. doi: 10.1128/mBio.02880-20 (PMC7773993; doi:10.1128/mBio.02880-20)
Supplement: TABLE S4 [file mBio.02880-20-st004.pdf]

Table S4. Atg24 in vitro PKA phosphorylation assay MS results

Peptide Sequence	Phosphorylation Site(s)	Localization Probability (%)	PKA Motif	Ascore	Ion Score	Charge
PE**[Sp]**DISRPTTSGTDADESLEYNRDTNGK	**S18**	**100**	No	27.16	82.36	3
QDPE**[Sp]**DISRPTTSGTDADESLEYNR	S18	90	No	12.63	91.66	3
PE**[Sp]**DI**[Sp]**RPTTSGTDADESLEYNR	**S18**; **S21**	**96**; **95**	No	18.19, 16.88	51.59	2
PE**[Sp]**DI**[Sp]**RP**[Tp]**TSGTDADESLEYNR	S18; S21; T24	86; 69; 34	No	9.22, 5.09, 0.00	42.48	3
PE**[Sp]**DISRPTTSGTDADE[Sp]LEYNR	**S18**; S33	**98**; **100**	No	22.36, 45.34	86.04	3
PESDI**[Sp]**RPTTSGTDADESLEYNR	S21	12	No	3.51	38.4	3
PESDI**[Sp]**RP**[Tp]**TSGTDADESLEYNR	S21; T24	20; 20	No	0.00, 0.00	21.34	3
PESDISRPT**[Tp]**SGTDADESLEYNR	T25	83	No	12.04	80.13	3
PESDISRPTT**[Sp]**G**[Tp]**DADESLEYNR	S26; T28	78; 15	No	9.17, 0.00	30.2	3
**[Tp]**DADESLEYNR	**T28**	**100**	No	49.39	22.12	2
PESDISRPTTSGTDADE**[Sp]**LEYNR	**S33**	**100**	No	19.04	79.9	3
RM**[Sp]**SVHED	**S47**	**100**	**Yes**	26.38	11.12	2
RM**[Sp]**SVHEDPPQAGPLADAVDLAGIGDGVLECR	S47	64	**Yes**	2.52	88.96	4
MS**[Sp]**VHEDPPQAGPLADAVDLAGIGDGVLECR	S48	50	No	0	72.56	3
S**[Sp]**VHEDPPQAGPLADAVDLAGIGDGVLECR	S48	50	No	0	35.43	3
RE**[Sp]**DLEADYNDLATQFR	**S266**	**100**	**Yes**	140.64	74.12	2
DSLAANPSSYYASNPLTS**[Sp]**PASFIR	S379	81	No	8.14	61.61	2
